# *Xifeng Zhichou* decoction mitigates tic disorder on juvenile rats by regulating neuroinflammation and neurotransmitter homeostasis: dual modulation of Nr4a2 and gut microbiota

**DOI:** 10.1186/s13020-026-01464-3

**Published:** 2026-07-08

**Authors:** Ruolan Wu, Jinlan Peng, Mingyue Zhang, Qing He, Jin Luo, Qinqiang Long, Xue Xiao, Shasha Li, Lisheng Wan

**Affiliations:** 1 The Second Clinical College, Guangzhou University of Chinese Medicine, No. 55, Inner Ring Road, Higher Education Mega Center, Panyu District, Guangzhou, 510006 Guangdong China; 2 Shenzhen Children’s Hospital, No. 7019, Yitian Road, Futian District, Shenzhen, 518000 Guangdong China; 3 Institute of Chinese Medicine, Guangdong Pharmaceutical University, No. 280, Outer Ring East Road, Higher Education Mega Center, Panyu District, Guangzhou, 510006 Guangdong China

**Keywords:** *Xifeng Zhichou* decoction, Tic disorder, Neuroinflammation, Neurotransmitter homeostasis, Nr4a2, Bile acid metabolism, Molecular mechanism

## Abstract

**Background:**

*Xifeng Zhichou* decoction (XFZCD), a modified formulation of Chinese medicine, demonstrates significant clinical efficacy in treatment of tic disorder (TD). However, its efficacy and therapeutic mechanisms remain insufficiently characterized.

**Objective:**

This study aims to comprehensively evaluate the anti-TD effect of XFZCD in a rat model, and to elucidate the underlying mechanism.

**Methods:**

Ultra-performance liquid chromatography–mass spectrometry (UPLC-MS) was used to quality analyze of XFZCD, while network pharmacology was employed to predict core compounds and potential mechanisms. A juvenile TD rat model was established via intraperitoneal injection of 3,3-iminodipropionitrile, and the therapeutic effects of XFZCD were evaluated using behavioral tests, cytokine assays, and immunohistochemistry. Transcriptomics and 16S rRNA sequencing were employed to elucidate the mechanisms by investigating regulatory gene and gut-brain axis. Western blot (WB) and enzyme-linked immunosorbent assay were utilized to focus and verify the potential mechanisms. Untargeted metabolomics was conducted to confirm the crucial pathway from the perspective of terminal metabolites. Furthermore, Nr4a-targeted siRNA knockdown in Lipopolysaccharides (LPS)-challenged SH-SY5Y cells was performed to verify the core regulatory role of Nr4a2 in XFZCD-mediated neuroprotection.

**Results:**

The UPLC-MS analysis identified 66 compounds in XFZCD. Network pharmacology highlighted serotonergic and dopaminergic synapses, and JAK-STAT signaling in enrichment analysis, suggesting XFZCD treats TD by regulating neuroinflammation and neurotransmitter homeostasis. XFZCD significantly mitigated tics, abnormal motor behavior, and anorexia in rat models, while also attenuating striatal neuroinflammation and oxidative stress, thereby exhibiting a substantial therapeutic effect on TD. Transcriptomic analysis identified Nr4a2 as a pivotal regulatory gene, which exhibited strong binding affinity with the core components identified via network pharmacology. 16S rRNA sequencing revealed that XFZCD modulated intestinal function by restoring the *Firmicutes*/*Bacteroidetes* ratio. WB and ELISA experiments confirmed that XFZCD could suppress inflammatory responses and restore central neurotransmitter homeostasis. Untargeted metabolomics indicated XFZCD reversed TD-caused abnormal bile acid metabolism, which was closely correlated with gut microbial remodeling. Cellular experiments confirmed that Nr4a2 silencing largely abrogated XFZCD’s inhibitory effects on neuroinflammation and its regulatory function on neurotransmitter balance.

**Conclusion:**

XFZCD demonstrates significant efficacy in the treatment of TD by inhibiting inflammation and balancing neurotransmitters through dual modulation of Nr4a2 and intestinal flora. Notably, the regulation of Nr4a2 and bile acid metabolism emerges as a promising therapeutic avenue for the treatment of TD.

**Supplementary Information:**

The online version contains supplementary material available at 10.1186/s13020-026-01464-3.

## Introduction

Tic disorder (TD) is a complex neurodevelopmental disorder that is highly prevalent in children [1]. A national epidemiological survey in China indicated that the prevalence of TD among school-aged children and adolescents aged 6–16 years was 2.46% [2]. TD patients often have sudden, rapid, repetitive and rhythmic motor tics and/or vocal tics [3, 4], which lead to academic, social and mental troubles and seriously affect the quality of life of children and their families. At present, α2-adrenergic agonists and antipsychotics[5, 6] used in the clinical treatment of TD are limited in clinical application because of their side effects such as weight gain, drowsiness, extrapyramidal symptoms and metabolic abnormalities [7, 8]. Therefore, there is an urgent need to develop safe and effective pharmacological treatments for TD.

According to TCM theory, the pathogenesis of TD is intricately linked to internal stirring of *liver wind* [9, 10]. The therapeutic strategy of *calming the liver* to *extinguish wind* has shown promising clinical outcomes, with minimal adverse reactions reported [11, 12]. XFZCD is a distinctive formulation derived from classical TCM prescriptions, developed at Shenzhen Children’s Hospital through extensive clinical practice. Clinically, it has proven robust clinical efficacy in reducing the frequency of tics in TD children and improving associated indicators, including the Yale Global Tic Severity Scale (YGTSS) score. However, the inadequacy of systematic research and unclear mechanism hinder its standardized development and broader clinical application.

In this study, the components of XFZCD were qualitatively analyzed using UPLC-MS to illustrate the chemical constituents and network pharmacology was performed to predict core components and multi-target therapeutic features. TD model was established using juvenile rats to simulate the clinical characteristics observed in TD children. The effects of XFZCD were systematically evaluated through behavioral manifestations, neuroinflammation, oxidative stress, and microglial activation. Mechanistic insights were explored using transcriptomics and 16S rRNA sequencing, focusing on regulatory gene and gut-brain axis. Further investigations, including metabolomics, ELISA, WB, and siRNA-mediated Nr4a2 knockdown in LPS-stimulated SH-SY5Y cells were performed to validate the molecular mechanisms. This study provides mechanistic evidence linking Nr4a2 and gut microbiota modulation to neuroinflammatory and neurotransmitter regulatory in TD, thereby supporting XFZCD as a promising multi-target therapeutic candidate for TD.

## Materials and methods

### Qualitative analysis of XFZCD

XFZCD consisted of *Rehmannia glutinosa* Libosch. (Dihuang), *Paeonia lactiflora* Pall. (Baishao), *Ostrea gigas* Thunberg (Muli), *Uncaria rhynchophylla* (Miq.) Miq. ex Havil. (Gouteng), *Hyriopsis cumingii* (Lea) (Zhenzhumu), *Bombyx mori* Linnaeus (Jiangcan), *Gastrodia elata* Bl. (Tianma), *Curcuma wenyujin* Y. H. Chen et C. Ling (Yujin), *Acorus tatarinowii* Schott (Shichangpu), *Polygala tenuifolia* Willd. (Yuanzhi), *Chaenomeles speciosa* (Sweet) Nakai (Mugua). All herbs were purchased from *Guangdong Provincial Hospital of Traditional Chinese Medicine*. The detailed dosage of each herbal component in the clinical prescription of XFZCD is provided in Table 1. Muli and Zhenzhumu were decocted in eight times total weight of water for one hour first. The remaining herbs were added and decocted for another two hours. The liquid was filtered and concentrated to 2 g/mL and stored at − 20 °C.

**Table 1:** Composition of XFZCD

Chinese name	Latin name of botanical plant	Part used	Formula composition ratio
Dihuang	*Rehmannia glutinosa* Libosch	Roots	2
Baishao	*Paeonia lactiflora* Pall	Roots	2
Muli	*Ostrea gigas* Thunberg	Shell	3
Gouteng	*Uncaria rhynchophylla* (Miq.) Miq. ex Havil	Root stems	2
Zhenzhumu	*Hyriopsis cumingii* (Lea)	Shell	3
Jiangcan	*Bombyx mori* Linnaeus	Stiff Silkworm	2
Tianma	*Gastrodia elata* Bl	Roots	1
Yujin	*Curcuma wenyujin* Y. H. Chen et C. Ling	Roots	2
Shichangpu	*Acorus tatarinowii* Schott	Roots	2
Yuanzhi	*Polygala tenuifolia* Willd	Roots	2
Mugua	*Chaenomeles speciosa* (Sweet) Nakai	Fruit	1

### Determination of XFZCD

Component analysis of XFZCD was conducted using UPLC (Waters, USA)—quadrupole time-of-flight mass spectrometer (Q-TOF-MS) (TripleTOF® 5600; AB SCIEX, USA) controlled by Analyst® TF 1.6 software. Chromatographic separation was achieved using ultrapure water (mobile phase A) and acetonitrile (mobile phase B) through gradient elution with an Acquity UPLC BEH C_18_ column (2.1 mm × 100 mm, 1.7 μm) maintained at 35 °C. The injection volume was 2 μL. The gradient conditions for chromatographic separation were established as follows: 0–3 min, 2% B; 4–9 min, 15% B; 16 min, 45% B; 28–30 min, 98% B.

The MS detection was carried out both in positive and negative ion mode with settings were as followed: the source temperature stayed at 500 °C and the spray voltage was adjusted to 5500 V (+) or 4500 V (−). The collision energy was set to 10 V for TOF MS and 40 ± 20 V for Product Ion. For scanning compounds, the instrument was set to detect mass ranging from 100 to 2000 m/z (TOF MS) and 50 to 1500 m/z (Product Ion), utilizing Information-Dependent Acquisition (IDA) as the data acquisition mode.

The ingredients of each constituent herb in XFZCD were sourced from the TCMSP (www.tcmsp-e.com/database), TCMID (www.bidd.group/TCMID/), BAT-MAN (bionet.ncpsb.org.cn/batman-tcm/index.php), HERB (http://herb.ac.cn/), ChemSpider (www.chemspider.com/) databases. Compounds were identified by utilizing critical mass spectrometry data including m/z, fragmentation patterns, and isotopic distribution with PeakView 1.2 software.

### Network pharmacology

The potential targets of the identified XFZCD compounds were predicted using Swiss Target Prediction (https://swisstargetprediction.ch/). Therapeutic targets for TD were obtained from Genecards (https://www.genecards.org/), OMIM (https://www.omim.org/), and Therapeutic Target (https://db.idrblab.net/ttd/). Common therapeutic targets were obtained by intersecting compound-related targets and disease-related targets. The compound-target-disease interaction network was constructed and visualized using Cytoscape software (v3.10.3). Topological analysis based on degree centrality was performed to screen core compounds of XFZCD, which were subsequently selected as candidate ligands for molecular docking. The top 20 targets ranked by degree value were submitted to the STRING database (https://cn.string-db.org/) to construct a protein–protein interaction (PPI) network, with the organism set to *Rattus norvegicus* and a confidence threshold of 0.15. Subsequently, Gene Ontology (GO) functional enrichment and Kyoto Encyclopedia of Genes and Genomes (KEGG) pathway enrichment analyses was conducted on the intersecting genes using Metascape (https://metascape.org/), and the results were visualized using Cytoscape.

### Animals and drug administrations

Male juvenile Sprague–Dawley rats (weight, 50–70 g; postnatal day 21–23) were obtained from *Guangdong Vital River Laboratory Animal Technology Co., Ltd.* (Foshan, China) (Animal quality certificate: 44007200127934). The animals were housed under a 12 h day/night cycle at room temperature (20–26 °C), 40%–70% relative humidity for the study and were given ad libitum access to water and food. The experiments were conducted under the full authorization from the *Ethics Committee of Guangdong Pharmaceutical University* (No. gdpulacspf2022400).

Upon arrival, all rats were acclimatized for 3 days before randomization and any experimental procedures. After acclimatization, rats were randomly divided into six groups (n = 12): control group (CON), model group (MOD), positive group (TIA, Tiapride hydrochloride 25 mg/kg/d. lot: DLY23B01, *Jiangsu Nhwa Pharmaceutical Co., Ltd.*), XFZCD low dose group (LZD, 4.58 g/kg/d), XFZCD medium dose group (MZD, 9.17 g/kg/d, clinical equivalent dose), and XFZCD high dose group (HZD, 18.3 g/kg/d). With the exception of the CON group, all rats were administered daily intraperitoneal injections of 3,3-Iminodipropionitrile (IDPN, lot: I829733, Macklin, China) at a dosage of 150 mg/kg for seven consecutive days. The CON group received an equivalent volume of saline solution. Following 28 days of drug intervention, all rats received a final drug administration 30 min prior to euthanasia using isoflurane. Samples of serum, feces, and brain tissue were collected and stored at − 80 °C for further analysis.

### Behavioral tests

Body weight, food, and water intake were monitored and recorded every three days throughout the study. Before undergoing behavioral testing, the rats were acclimated to the testing environment and handling procedures over a period of three consecutive days. Open field test (OFT) was conducted using computerized video tracking system (SMART 3.0) to evaluate general locomotor and exploratory activities. All recordings and movement trajectories were checked by trained investigators who were blinded to the group allocations. Data sets with incomplete recordings, tracking failures, evident trajectory artifacts, or inaccurate zone detection were excluded from the analysis. Each rat was continuously recorded for 210 s, during which the total distance traveled, average speed, and time spent in the central zone were measured.

Motor and stereotyped behaviors were independently assessed by two trained observers who were blinded to the group allocations in accordance with predefined criteria (Table 2). Tic-like behaviors induced by IDPN were characterized by repetitive, stereotyped, small rhythmic movements (e.g., head shaking, sniffing, grooming) that occur without postural imbalance or circling. A stereotyped behavior score ≥ 2 and a motor behavior score ≥ 2 was defined as criteria for successful model establishment following IDPN administration [13, 14]. Behaviors indicative of severe vestibular dysfunction, such as persistent head tilt, rolling, postural instability, marked ataxia, or impaired righting reflex, were excluded from scoring [15, 16]. Rats were housed individually in observation chambers under sound-attenuated and dark conditions. After habituating to the environment for 5 min, the behaviors of rats were recorded for 2 min.

**Table 2:** Motor behavior score and stereotype score criteria

Score	Motor behavior	Stereotype behavior
0	Quiet or normal activities	No-stereotyped exercise
1	Overexcitement	Rotation
2	Inquiry-based behavior increases	Head vertical movement
3	The weakened limbs, the range of exploration decreases	Head vertical movement and rotation, increasing repeating nasal smell
4	Crawling forward, the range of its motion is small circle	Head lateral pendulum with head vertical movement, sniffing, biting, and licking continuously

### Immunohistochemistry

Brain tissue sections underwent standard deparaffinization and hydration procedures, followed by a 50 min heat-induced antigen retrieval process. Subsequently, the sections were incubated in a 3% hydrogen peroxide solution for 25 min in the dark. The tissues were then blocked with 3% bovine serum albumin (BSA) for 30 min at ambient temperature. Following a washing step, the sections were incubated with a primary antibody against IBA1 (dilution 1:1000, Servicebio, China) in a humidified chamber overnight at 4 °C. After washing, sections were incubated with an HRP-conjugated IgG secondary antibody for 50 min at room temperature, followed by visualization with Diaminobenzidine for 45 s. Finally, sections were counterstained with hematoxylin for 1 min, dehydrated through graded ethanol, cleared in xylene, mounted with neutral resin, and examined under a light microscope to assess the morphology. For each section, three non-overlapping fields in the striatal region were selected for quantification. The IBA1 positive area was measured using ImageJ software and expressed as the percentage of positive staining area. Representative enlarged insets were added to show the morphological features of individual IBA1 positive microglia.

### Transcriptomic research

Total RNA was extracted from striatum tissue of the CON, MOD, and HZD groups for cDNA library construction and Illumina sequencing which was performed by *Baimake Biotechnology Co., Ltd. (China)*. After trimming raw reads, they were aligned to the reference genome (Ensembl Release 106), and gene expression was quantified as FPKM using RSEM software. Differentially expressed genes (DEGs) were identified with DESeq2 using thresholds of FC ≥ 2 and adjusted *P* < 0.05, with False Discovery Rate (FDR) correction for 5% false positives. KEGG pathway enrichment analysis was conducted via OmicShare.

### Molecular docking

Structural information for the core components and Nr4a2 was sourced from the PubChem and Protein Data Bank (PDB ID: 5Y41) databases, respectively. PyMOL 2.6.0 was employed to remove water and co-crystallized ligands, while GetBox Plugin was utilized to determine the docking pocket and grid box parameters. Docking simulations were conducted using AutoDock Vina (version 1.1.2), which assessed binding affinity based on docking binding energy, with lower energy values indicating stronger affinity. PyMOL visualized the docking conformations and protein–ligand interactions.

### 16S rRNA sequencing analysis

Fecal samples from CON, MOD, and XFZCD groups rats underwent 16S rRNA sequencing, performed by *Beijing Biomarker Technologies Co., Ltd*. High-quality sequences were clustered into Operational Taxonomic Units (OTUs) at 97% sequence similarity using USEARCH software (version 10.0), followed by a conservative filtration threshold of 0.005%. Alpha-diversity was assessed using the Shannon index, while beta-diversity was analyzed through unweighted pair-group method with arithmetic means (UPGMA) clustering based on Bray–Curtis dissimilarity matrices to visualize differences in community structure. The relative abundance of bacterial taxa was analyzed and visualized at both phylum and genus levels. 16S rRNA gene sequences were employed to predict functional pathways against the KEGG database using PICRUSt2. Spearman correlation analysis was performed to quantify the correlations between differential flora and peripheral bile acids as well as central inflammatory cytokines and neurotransmitters.

### Untargeted serum metabolomics

The serum samples of rats from the CON, MOD and HZD groups were chosen for metabolomics analysis. The serum was prepared by mixing with methanol at a 1:3 (v/v) ratio, and centrifuged at 13,000 rpm for 15 min at 4 °C. The supernatant was filtered by 0.22 μm filter membrane. The UPLC separation was performed using ultrapure water (phase A) and acetonitrile (phase B) for gradient elution with an Acquity UPLC BEH C18 column (2.1 mm × 100 mm, 1.7 μm) at 35 °C. Key source parameters were configured as follows: the collision energy was set to 10 V for TOF MS and 35 ± 20 V for Product Ion. The scan ranges of 100–500 m/z for TOF MS and 50–1500 m/z for Product Ion; ion source temperature at 600 °C; and spray voltage at 5500 V. The gradient conditions for chromatographic separation were established as follows: 0–2 min, 2% B; 5 min, 25% B; 10 min, 50% B; 18 min, 60% B; 30 min, 68% B; 33 min, 85% B; 38–50 min, 98%B.

Raw mass spectrometry data were initially processed using Progenesis QI version 2.4 (Waters, USA), which included background filtering, peak detection, integration, retention time correction and peak alignment to generate the data matrix. The metabolites were identified by matching accurate mass and tandem MS data with the human metabolome database (HMDB). A mass tolerance of 5 ppm was applied for both precursor and fragment ions. This processed data was then imported into SIMCA-P + (version 14.0) for principal components analysis (PCA) and partial least squares discrimination analysis (PLS-DA). Differential metabolites were subsequently screened from the PLS-DA model based on Variable Importance in the Projection (VIP) > 1 and *P* < 0.05 in statistical analysis. Pathway analysis of the differential metabolites was conducted using MetaboAnalyst (Version 6.0).

### Preparation of XFZCD-containing serum

Thirty male Sprague–Dawley rats (weight, 250–270 g; postnatal day 60–70) were procured from *Guangdong Vital River Laboratory Animal Technology Co., Ltd.* (Foshan, China, animal quality certificate number 44829700048693, Experimental facility certificate: 00463973). Following an acclimatization period, the rats were randomly assigned into blank serum group (n = 10) and XFZCD containing serum group (n = 20). The XFZCD group were orally administered XFZCD at 18.3 g/kg twice daily, while the control group was administered an equivalent volume of water. On the fourth day, blood was collected from the abdominal aorta 1 h post-administration using procoagulant-separation gel. The samples were centrifuged at room temperature for 10 min at 3000 rpm, and the supernatant was filtered through a 0.22 μm filter. The filtrate was inactivated at 56 °C for 30 min, and stored at − 20 °C for future use.

### Cell transfection and treatment

SH-SY5Y cells (iCell-h187) were divided into five groups: control (CON), LPS-stimulated model (LPS), XFZCD-containing serum treatment (XFZCD), Nr4a2 knockdown plus LPS model (KD), and Nr4a2 knockdown plus XFZCD-containing serum (KD + XFZCD). Both the LPS and XFZCD groups were transfected with negative control siRNA, while the KD and KD + XFZCD groups were transfected with Nr4a2 siRNA using the RFect V2 transfection reagent (*Changzhou Bio-generating Biotechnology Corp*). Subsequent to the treatment, cells were harvested for WB analysis to detect the expression of Nr4a2, IκBα, and the phosphorylation levels of NFκB, JAK1, and STAT3.

### ELISA detection

The striatum tissues of rats from all groups were collected to test TNFα (lot. 20250217A-0036B), IL-6 (lot no. 20250209A-0031B), glutathione peroxidase (GPx, lot no. 20250216A-2382B), superoxide dismutase (SOD, lot no. 20250216A-2102B) and malondialdehyde (MDA, lot no. 20250216A-3005B) levels. The cerebral cortex of rats from all groups were collected to test dopamine (DA, lot no.20251112A-1517B), glutamic acid (Glu, lot no.20251112A-3622B), Gamma-aminobutyric acid (GABA, lot no.20251112A-2506B) and serotonin (5-HT, lot no.20251112A-2819B) level. The cells were washed with pre-cooled PBS and lysed in RIPA buffer (P0013D, Beyotime, China), and the resultant lysates were collected for ELISA quantification of DA, Glu, GABA and 5-HT. All ELISA procedures strictly followed the manufacturer’s instructions (*Shanghai Enzyme-linked Biotechnology Co., Ltd., China*). Samples with insufficient volume, technical assay failure were excluded from the corresponding biochemical parameter.

### WB analysis

Rat striatum protein samples were separated by 8% SDS-PAGE and transferred to PVDF membranes (*Merck Millipore Co., Ltd*). Protein bands were visualized through enhanced chemiluminescence (ECL) detection and quantified by gray scale analysis using ImageJ software, with α‑Tubulin serving as the internal reference for normalization. All quantification data were calculated from multiple independent biological replicates for subsequent statistical evaluation.

The primary antibodies were Nurr1(Nr4a2) (sc-376984, Santa, 1:1000); p-JAK1 (Tyr1034, CST, 1:1000), JAK1 (6G4, CST, 1:1000), p-STAT3 (Tyr705, CST, 1:1000), STAT3 (D3Z2G, CST, 1:1000), p-NFκBp65 (S536, CST, 1:1000), NFκB (D14E12, CST, 1:1000), IκBα (L35A5, CST, 1:1000), and α-Tubulin(11H10, CST, 1:4000).

### Statistical analysis

All data were presented as the mean ± standard deviation and analyzed using Graphpad Prism (Version 10.1). The normality of continuous data was assessed via the Shapiro–Wilk test, and the comparisons between groups was assessed using one-way ANOVA and Kruskal–Wallis H test for normally distributed data and non-normally distributed data respectively. One-way ANOVA was conducted for data with equal variances, and Welch's ANOVA was employed for data with unequal variances. A *P* value of less than 0.05 was considered statistically significant, whereas a *P* value of less than 0.01 indicated highly significant differences. All probability values were computed as two-tailed.

## Results

### Chemical analysis of XFZCD

**Fig. 1:**
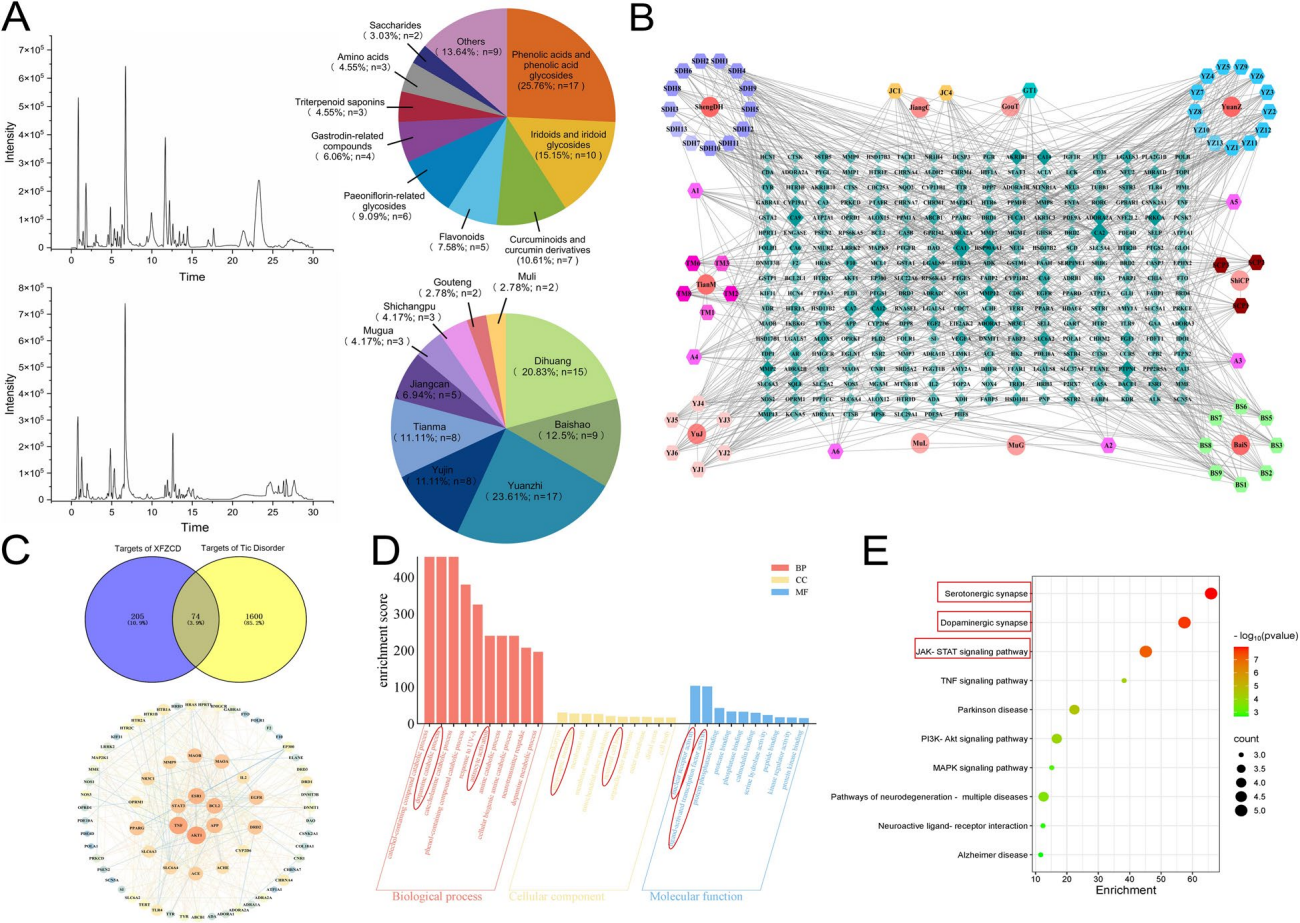
Chemical characterization and network pharmacology analysis of XFZCD. **A** UPLC-MS analysis of XFZCD, including base peak chromatograms in positive- and negative-ion modes, chemical classification, and herb-source attribution of the identified compounds; **B** Herb-component-target network of XFZCD (ShengDH: Dihuang; TianM: Tianma; YuJ: Yujin; MuL: Muli; MuG: Mugua; BaiS: Baishao; ShiCP: Shichangpu; YuanZ: Yuanzhi; GouT: Gouteng; JiangC: Jiangcan; The Compound ID was listed in the supplementary table S1); **C** Venn diagram of XFZCD against TD and PPI network of the common targets; **D** GO enrichment analysis of the common targets; **E** KEGG pathway enrichment analysis of the common targets

A total of 66 compounds were identified and characterized in XFZCD by UPLC-MS in positive- and negative-ion modes. Base Peak Chromatogram (BPC) were presented in Fig. 1A. The identified compounds were categorized by chemical subclass and corresponding original medicinal herbs (Fig. 1A). Chemically, phenolic acids and its glycosides constituted the predominant fraction, followed by iridoids, flavonoids, paeoniflorins, and so on. Source tracing showed most characteristic compounds originated from Yuanzhi, Dihuang, Baishao, Yujin, and Tianma. Detailed qualitative information was listed in Supplementary materials Table S1.

### Key components and possible targets of XFZCD for TD treatment

The herb-component-target network was constructed (Fig. 1B), and the top 20 core compounds of XFZCD were screened on the base of degree value (Table S2). Intersection of compound-targets and TD-related disease targets yielded 74 overlapping genes, and PPI network analysis highlighted AKT1, TNF and STAT as critical targets (Fig. 1C). GO functional enrichment analysis revealed significant enrichment in dopamine metabolism, nuclear receptor activity, and neurotransmitter regulation (Fig. 1D). KEGG pathway analysis underscored the involvement of serotonergic, dopaminergic synapses and the JAK–STAT signaling pathway (Fig. 1E). These findings suggest that XFZCD may ameliorate TD by modulating neurotransmitter homeostasis and neuroinflammatory responses.

### XFZCD improved behavioral indicators of TD rats

**Fig. 2:**
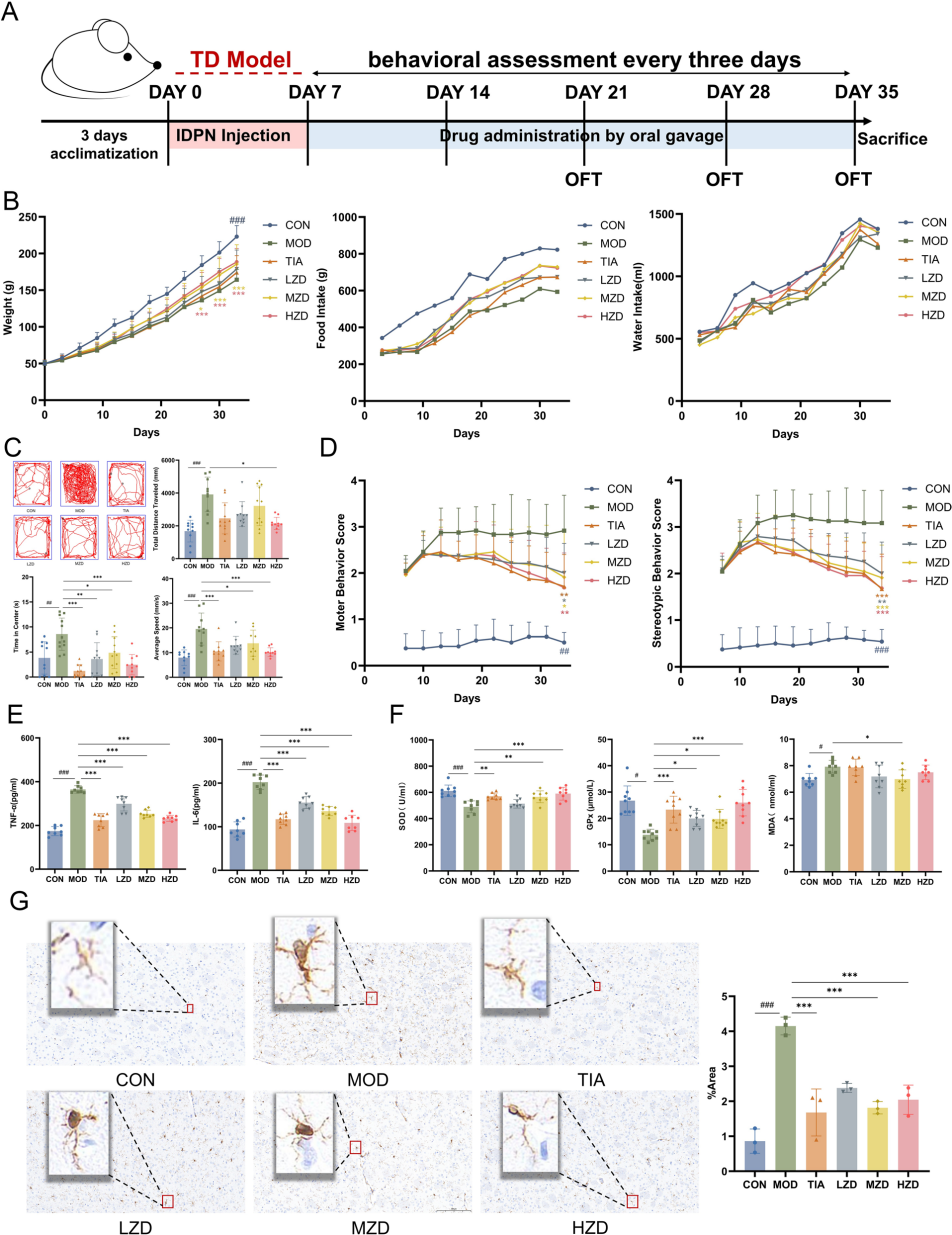
Effects of XFZCD on tic-like behaviors, neuroinflammation, oxidative stress, and microglial activation in TD rats. **A** Experimental timeline; **B** Changes in body weight, food intake, and water intake during the experimental period; **C** Representative movement trajectories in the open-field test and quantitative analysis of total distance traveled, time spent in the center area, and average speed (total distance traveled, TIA n = 12, MZD n = 11, and other groups n = 10; time in central zone, MOD n = 12, MZD n = 11, and other groups n = 10; average speed, MZD n = 9 and other groups n = 10); **D** Motor behavior score and stereotypic behavior score in each group (n = 12); **E** Striatal levels of the pro-inflammatory cytokines (n = 8 for TNFα and IL-6); **F** Striatal oxidative stress markers (n = 9 for GPx, SOD and MDA); **G** Representative immunohistochemical images of IBA1-positive microglia in the brain tissue (scale bar = 200 μm, n = 3) and the corresponding quantitative analysis of the IBA1-positive area (% area). CON: control group; MOD: Tic disorder model group; TIA: positive control group; LZD: *Xifeng Zhichou* Decoction low-dose administration group; MZD: *Xifeng Zhichou* Decoction medium-dose administration group; HZD: *Xifeng Zhichou* Decoction high-dose administration group. ^#^*P* < *0.05*, ^##^*P* < 0.01, ^###^*P* < 0.001 vs. CON group; *P < 0.05, **P < 0.01, ***P < 0.001 vs. MOD group; no symbol indicates no significant difference (*P* > 0.05)

The experimental design is illustrated in Fig. 2A. As shown in Fig. 2B, the MOD group exhibited reduced body weight gain and decreased food and water intake compared to the CON group (*P* < 0.001). Following treatment, the MZD and HZD groups demonstrated increased body weight (*P* < 0.001, vs MOD) and improved intake relative to the MOD group. No significant differences were observed between the TIA and MOD groups, potentially due to the appetite-suppressing effects of TIA.

The OFT (Fig. 2C) revealed that the MOD group exhibited hyperactivity relative to the CON group, evidenced by a significantly increased total travel distance, time spent in the central area and average movement speed (*P* < 0.01, vs CON). Both TIA and XFZCD interventions markedly shortened time in center and average speed (*P* < 0.001, vs MOD) and induced a numerical decline in total distance traveled, with this reduction achieving statistical significance only in HZD group (*P* < 0.05, vs MOD). These results demonstrate that both TIA and XFZCD effectively alleviate anxiety and excessive spontaneous activity in IDPN-induced rats, and HZD produces the optimal therapeutic effect among all XFZCD-treated cohorts.

The scores of motor behavior and stereotype behavior (Fig. 2D) showed that MOD rats exhibited remarkably increased levels of excessive locomotion (*P* < 0.01) and heightened rigidity compared to the CON group (*P* < 0.001). Administration of either TIA or XFZCD significantly decreased the above behavioral scores (*P* < 0.01, vs MOD), and HZD exerted an anti-stereotypy efficacy comparable to TIA. These findings suggest that XFZCD can effectively ameliorate convulsion, abnormal motor behavior in TD rats, with its therapeutic efficacy being comparable to that of TIA.

### XFZCD attenuated neuroinflammation, oxidative stress, and microglial activation in TD rats

ELISA was conducted to detect the indicators of inflammation and oxidative stress in rat striatum. As illustrated in Fig. 2E, TNFα and IL-6 concentrations were markedly elevated in the MOD group compared to the CON groups (*P* < 0.001), indicating pronounced neuroinflammation in TD rats. Following treatment with TIA or XFZCD, there was a significant reduction in proinflammatory cytokines (*P* < 0.001, vs MOD), with the TIA and the HZD groups exhibiting the most pronounced effects. Notably, the efficacy of XFZCD in reducing inflammatory factors was dose-dependent. The assessment of oxidative stress markers revealed a significant decrease in the activity of GPx and SOD, alongside a substantial increase in MDA levels in the MOD group, which differed significantly from the CON group (Fig. 2F, *P* < 0.05), suggesting an abnormal oxidative stress state in TD rats. TIA and XFZCD exerted regulatory effects on oxidative stress indices in the rat striatum, with GPx and SOD showing the most significant changes (*P* < 0.01, vs MOD), whereas MDA was significantly reduced only in the MZD group compared with the MOD group (*P* < 0.05), without significant difference observed in the HZD group. These findings demonstrate that XFZCD effectively mitigates neuroinflammation and oxidative stress damage in brain of TD rats.

In addition, IBA1 immunohistochemistry was performed on brain tissue sections to provide in-situ morphological evidence of microglial activation (Fig. 2G). The specific positive staining was distinctly localized to the cytoplasm and processes of microglia. In contrast to the typical resting, ramified microglia observed in the CON group, the microglia in the MOD group were highly activated, exhibiting an amoeboid shape with hypertrophic cell bodies, thickened processes, and increased cell density. Interventions with XFZCD and TIA effectively reversed these morphological changes, suppressing microglial overactivation (*P* < 0.001, vs MOD).

### XFZCD regulated gene expression in TD rats

**Fig. 3:**
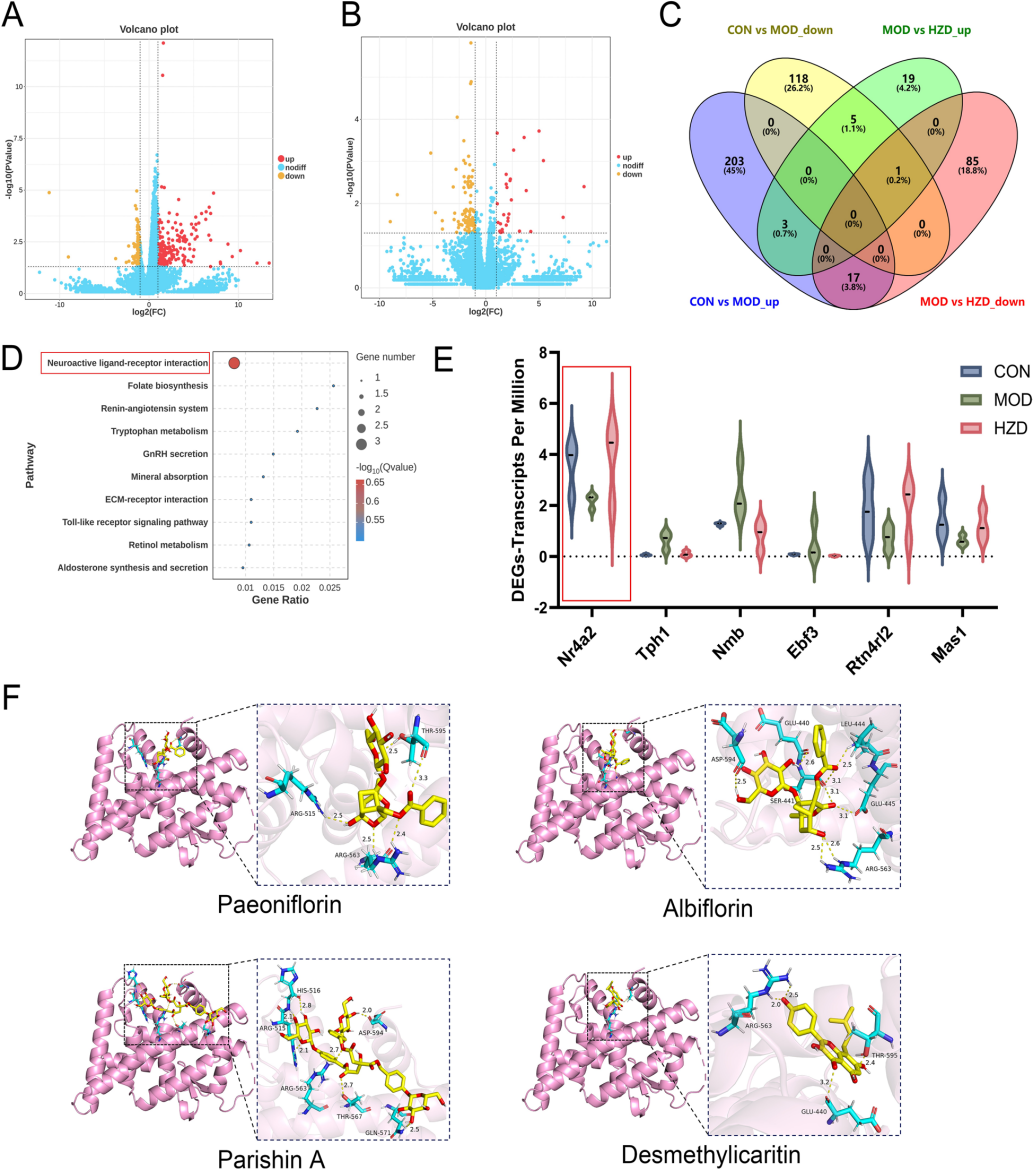
Transcriptomic analysis of striatal tissues in TD rats (n = 3). **A** Volcano plot of differentially expressed genes (DEGs) in the CON vs. MOD comparison; **B** Volcano plot of DEGs in the MOD vs. HZD comparison; **C** KEGG pathway enrichment analysis of the common DEGs; **D** Venn diagram showing the overlap among dysregulated genes in the two comparisons; **E** Violin plots of TPM values for selected DEGs in the CON, MOD, and HZD groups; **F** Representative docking conformations and interaction modes of the top four compounds, including paeoniflorin, albiflorin, parishin A, and desmethylicaritin, with Nr4a2. CON: control group; MOD: Tic disorder model group; HZD: *Xifeng Zhichou* Decoction high-dose administration group

Transcriptomic analysis of rat striatal tissues was performed to investigate gene expression patterns. Differential expression analysis using Volcano plots (Fig. 3A, B) screened common DEGs in the comparisons between MOD versus CON and HZD versus MOD, revealing 22 overlapping DEGs significantly modulated by XFZCD (Fig. 3C). KEGG pathway enrichment analysis of these DEGs highlighted pathways associated with neuroprotection and neuroinflammation, with the ligand-receptor interaction pathway being the most enriched (Fig. 3D).

Among the 22 DEGs, XFZCD reversed the overexpression of 17 genes and suppressed the expression of 5 genes, within Nr4a2, Tph1, Nmb, Ebf3, Rtn4rl2, and Mas1 are implicated in neurological regulation. Nr4a2 exhibited the most significant alteration, with significant downregulation observed in the MOD group compared to the CON group, and high-dose of XFZCD restored its expression to normal levels (Fig. 3E).

Molecular docking simulations were performed to assess the binding interactions between Nr4a2 and the top 20 core compounds of XFZCD. The calculated binding energies ranged from − 5.6 to − 8.0 kcal/mol (Table S2), confirming favorable binding affinity between the core ingredients and Nr4a2. Representative binding poses of paeoniflorin, albiflorin, parishin A and desmethylicaritin against Nr4a2 were visualized using PyMOL and are displayed in Fig. 3F. These results provided direct computational evidence that XFZCD directly target Nr4a2 to exert anti-TD effects.

### XFZCD altered the structure and diversity of gut microbiota

**Fig. 4:**
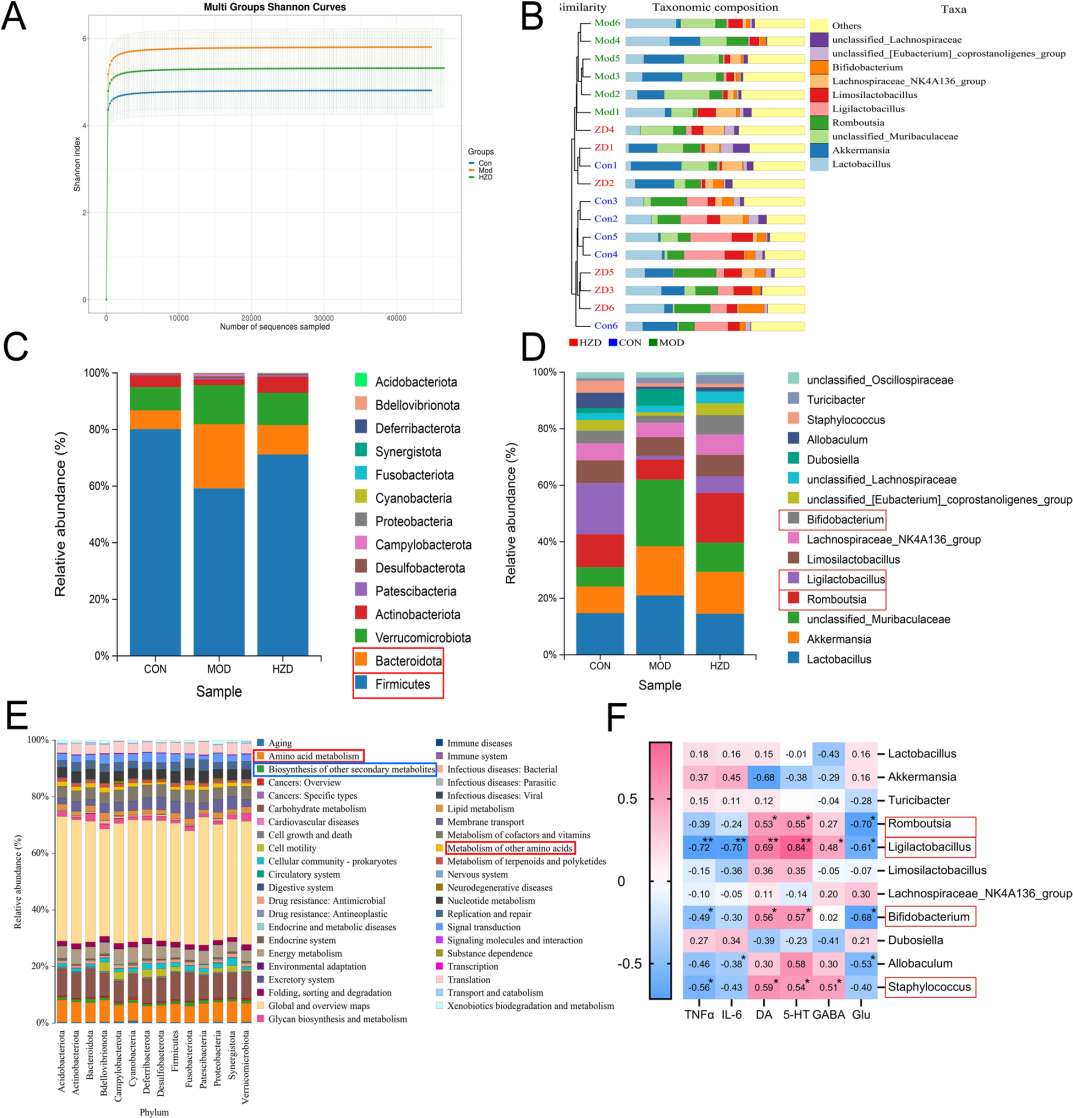
16S rRNA gene sequencing analysis of the gut microbiota (n = 6). **A** Alpha-diversity analysis based on the Shannon index; **B** UPGMA clustering analysis; **C** Relative abundance of bacterial communities at the phylum level; **D** Relative abundance of bacterial communities at the genus level; **E** Bar plot of predicted metabolic pathways; **F** Spearman correlation heatmap between differential gut microbiota and inflammatory cytokines/neurotransmitter. *P < 0.05, **P < 0.01; no symbol indicates no significant difference (*P* > 0.05). CON: control group; MOD: Tic disorder model group; HZD: *Xifeng Zhichou* decoction high dose administration group

The Shannon index revealed altered alpha diversity in MOD group compared to CON group. Following XFZCD treatment, the Shannon index in the HZD group tended to shift toward the CON level (Fig. 4A). The UPGMA clustering of beta diversity, based on Bray–Curtis dissimilarity, revealed distinct community structures among the groups (Fig. 4B). The MOD group exhibited the greatest divergence from the CON group, whereas the HZD group clustered closely with the CON group.

Phylum-level analysis indicated that IDPN modeling altered the relative abundance of dominant bacterial phyla, characterized by a decrease in *Firmicutes* and an increase in *Bacteroidetes* in MOD group compared to the CON group. XFZCD intervention partially ameliorated these alterations by increasing the abundance of *Firmicutes* and decreasing *Bacteroidetes*, aligning them more closely with the CON pattern (Fig. 4C). At the genus level, the relative abundances of *Ligilactobacillus*, *Bifidobacterium* and *Romboutsia* were diminished in MOD group compared to CON group. Intervention with XFZCD increased the abundances of these genera towards the levels observed in the CON group, suggesting a partial restoration of microbial alterations at the genus level (Fig. 4D). Functional predictions utilizing PICRUSt2 indicated potential variations in microbial functional profiles across the groups, with predicted pathways predominantly associated with amino acid metabolism and the biosynthesis of secondary metabolites (Fig. 4E).

Spearman correlation analysis demonstrated that *Ligilactobacillus*, *Staphylococcus, Bifidobacterium* and *Romboutsia* were negatively correlated with pro-inflammatory cytokines (TNF-α, IL-6) and the excitatory neurotransmitter (Glu), while exhibiting positive correlations with DA, 5-HT, and GABA (Fig. 4F). Among these associations, the correlations involving *Ligilactobacillus* achieved statistical significance (*P* < 0.05). These findings suggest that XFZCD induced changes in the abundance of these gut taxa are closely associated with the amelioration of neuroinflammation and the restoration of neurotransmitters.

### XFZCD rectifies systemic metabolic disturbances

**Fig. 5:**
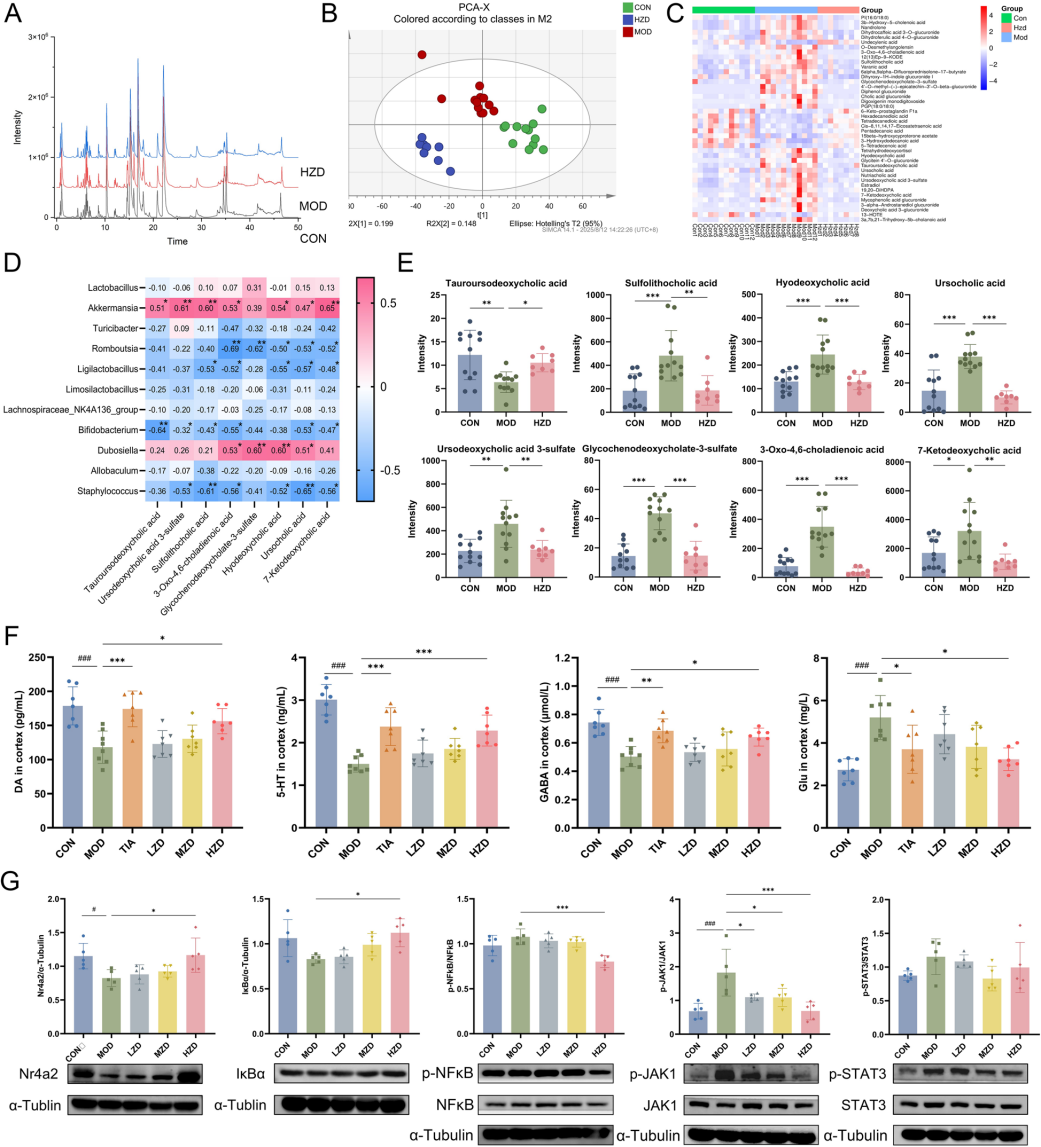
Validation of the effects of XFZCD on bile acid metabolism, neurotransmitter homeostasis, and Nr4a2 signaling in rats with TD. **A** Base peak chromatogram of rat serum metabolites (CON n = 12, MOD n = 12, HZD n = 8); **B** Principal component analysis (PCA) score plot of samples acquired in positive-ion mode; **C** Heatmap showing the relative abundance of differential metabolites; **D** Spearman correlation heatmap between differential gut microbiota and bile acids; **E** Relative levels of principal bile acids; **F** Effects of XFZCD and TIA on the levels of dopamine (DA), 5-hydroxytryptamine (5-HT), gamma-aminobutyric acid (GABA), and glutamate (Glu) (n = 8 for the MOD group and n = 7 for the other groups); **G** Effects of XFZCD on the protein expression of Nr4a2, IκBα, p-NFκB/NFκB, p-JAK1/JAK1, and p-STAT3/STAT3 (n = 5). CON: control group; MOD: Tic disorder model group; TIA: positive control group; LZD: *Xifeng Zhichou* Decoction low-dose administration group; MZD: *Xifeng Zhichou* Decoction medium-dose administration group; HZD: *Xifeng Zhichou* Decoction high-dose administration group. ^#^*P* < 0.05, ^##^*P* < 0.01, ^###^*P* < 0.001 vs. CON group; *P < 0.05, **P < 0.01, ***P < 0.001 vs. MOD group; no symbol indicates no significant difference (*P* > 0.05)

To mitigate the impact of confounding factors, untargeted metabolomics analysis was performed on serum samples from CON, MOD and HZD groups were analyzed using UPLC-Q/TOF MS (Fig. 5A). PCA demonstrated distinct clustering and separation among the groups, indicating significant metabolic disturbances in TD rats and a modulatory effect of XFZCD on the metabolomic profile (Fig. 5B). Metabolites with a VIP score exceeding 1.5 in PLS-DA and a *P*-value less than 0.05 in ANOVA were selected for further analysis. Finally, 42 differential metabolites were identified and are detailed in Table 3. A heat map illustrating their intensity is depicted in Fig. 5C. Bile acid and its derivatives, including Tauroursodeoxycholic acid (TUDCA), Sulfolithocholic acid, Hyodeoxycholic acid, Ursocholic acid, Ursodeoxycholic acid 3-sulfate, Glycochenodeoxycholate-3-sulfate, 3-Oxo-4,6-choladienoic acid, 7-Ketodeoxycholic acid, were identified as primary metabolites.

**Table 3:** Differential metabolites

HMDB	Name	m/z	RT (min)	ANOVA (p)	VIP
HMDB0062582	PI (34:0)	839.56	10.30	0.023	1.15
HMDB0000308	3b-Hydroxy-5-cholenoic acid	375.29	10.54	0.001	1.27
HMDB0002725	Nandrolone	275.20	13.47	0.001	1.07
HMDB0041720	Dihydrocaffeic acid 3-O-glucuronide	376.12	3.99	0.004	1.22
HMDB0041723	Dihydroferulic acid 4-O-glucuronide	390.14	4.52	0.017	1.14
HMDB0033724	Undecylenic acid	202.18	6.75	0.003	1.03
HMDB0004629	O-Desmethylangolensin	259.10	7.10	< 0.001	1.32
HMDB0000476	3-Oxo-4,6-choladienoic acid	371.26	9.22	0.004	1.03
HMDB0013623	12(13) Ep-9-KODE	309.21	13.48	< 0.001	1.18
HMDB0000907	Sulfolithocholic acid	455.25	12.57	0.002	1.30
HMDB0002195	Varanic acid	481.32	8.45	0.003	1.50
HMDB0060797	6alpha,9alpha-Difluoroprednisolone-17-butyrate	511.21	10.53	0.010	1.32
HMDB0059997	Dihyroxy-1H-indole glucuronide I	324.07	1.94	0.002	1.33
HMDB0002497	Glycochenodeoxycholate-3-sulfate	528.26	9.06	0.018	1.03
HMDB0029180	4'-O-methyl-(-)-epicatechin-3'-O-beta-glucuronide	493.13	4.99	< 0.001	1.21
HMDB0059998	Diphenol glucuronide	285.06	1.49	0.001	1.16
HMDB0002577	Cholic acid glucuronide	583.31	8.76	0.003	1.26
HMDB0060709	Digoxigenin monodigitoxoside	565.30	8.86	< 0.001	1.07
HMDB0013504	PGP (36:0)	903.54	9.20	0.001	1.31
HMDB0002886	6-Keto-prostaglandin F1a	369.23	8.07	0.015	1.07
HMDB0000672	Hexadecanedioic acid	285.21	10.67	< 0.001	1.26
HMDB0000872	Tetradecanedioic acid	257.18	9.50	0.001	1.11
HMDB0002177	Cis-8,11,14,17-Eicosatetraenoic acid	303.23	28.19	0.003	1.13
HMDB0000826	Pentadecanoic acid	287.22	11.62	0.001	1.08
HMDB0060708	15beta-hydroxycyproterone acetate	431.16	25.05	< 0.001	1.23
HMDB0000387	3-Hydroxydodecanoic acid	215.16	10.66	0.001	1.22
HMDB0000499	5-Tetradecenoic acid	227.20	11.38	0.007	1.10
HMDB0005972	Tetrahydrodeoxycortisol	351.25	11.43	0.003	1.14
HMDB0000733	Hyodeoxycholic acid	410.33	12.48	0.011	1.16
HMDB0041740	Glycitein 4'-O-glucuronide	461.11	5.35	< 0.001	1.01
HMDB0000874	Tauroursodeoxycholic acid	500.30	7.34	0.002	1.12
HMDB0000917	Ursocholic acid	426.32	8.52	0.034	1.13
HMDB0000467	Nutriacholic acid	391.28	9.19	0.006	1.11
HMDB0002642	Ursodeoxycholic acid 3-sulfate	471.24	9.46	0.001	1.40
HMDB0000151	Estradiol	317.18	12.04	< 0.001	1.41
HMDB0010214	19,20-DiHDPA	361.24	16.57	< 0.001	1.14
HMDB0000391	7-Ketodeoxycholic acid	451.27	9.40	0.011	1.20
HMDB0060634	Mycophenolic acid glucuronide	495.15	4.17	0.021	1.30
HMDB0010339	3-alpha-Androstanediol glucuronide	467.26	8.96	0.007	1.16
HMDB0013192	3a,7b,21-Trihydroxy-5b-cholanoic acid	469.28	9.35	0.010	1.16
HMDB0002596	Deoxycholic acid 3-glucuronide	567.32	8.86	< 0.001	1.15
HMDB0010203	13-HOTE	293.21	13.96	0.001	1.21

Spearman correlation analysis uncovered prominent correlations between altered gut microbial genera and differential bile acid metabolites (Fig. 5D). *Akkermansia* and *Dubosiella* showed positively correlations with most detected bile acid derivatives, while *Romboutsia*, *Ligilactobacillus*, *Bifidobacterium* and *Staphylococcus* were inversely associated with these bile acid molecules. The relative abundances of eight principal bile acids were presented in Fig. 5E. Compared with the CON group, the MOD group exhibited a significant reduction in the serum level of TUDCA, alongside marked elevations in the levels of seven other bile acids (*P* < 0.05, vs CON). XFZCD intervention effectively reversed these aberrant changes, restoring the bile acid profile toward the healthy CON group state (*P* < 0.05, vs MOD). These findings indicate that the therapeutic efficacy of XFZCD against TD is closely associated with the normalization of systemic bile acid metabolism.

### XFZCD restores neurotransmitter homeostasis and neuroinflammation

The analysis of cortical neurotransmitters indicated that the MOD group exhibited significantly decreased concentrations of DA, 5-HT, and GABA, along with marked elevated Glu levels, in comparison to the CON group (*P* < 0.001, Fig. 5F). Administration of XFZCD effectively ameliorated these pathological changes by significantly restoring DA, 5-HT, and GABA levels while reducing Glu levels, with all alterations demonstrating statistical significance relative to the MOD group (*P* < 0.05).

In the striatum (Fig. 5G), the MOD group exhibited markedly downregulated Nr4a2 expression (*P* < 0.05) and elevated phosphorylated JAK1 level compared with the CON group (*P* < 0.001). Although IκBα expression declined and the phosphorylation levels of NFκB and STAT3 increased in the MOD group, these alterations failed to reach statistical significance. (*P* = 0.08, 0.38, 0.32 respectively). Conversely, high dose XFZCD treatment significantly upregulated the expression of Nr4a2 and IκBα and inhibited the phosphorylation of NFκB and JAK1 (*P* < 0.05, vs MOD), thereby mitigating the activation of pro-inflammatory signaling pathways.

### Nr4a2 knockdown blocked the anti-TD effect of XFZCD

**Fig. 6:**
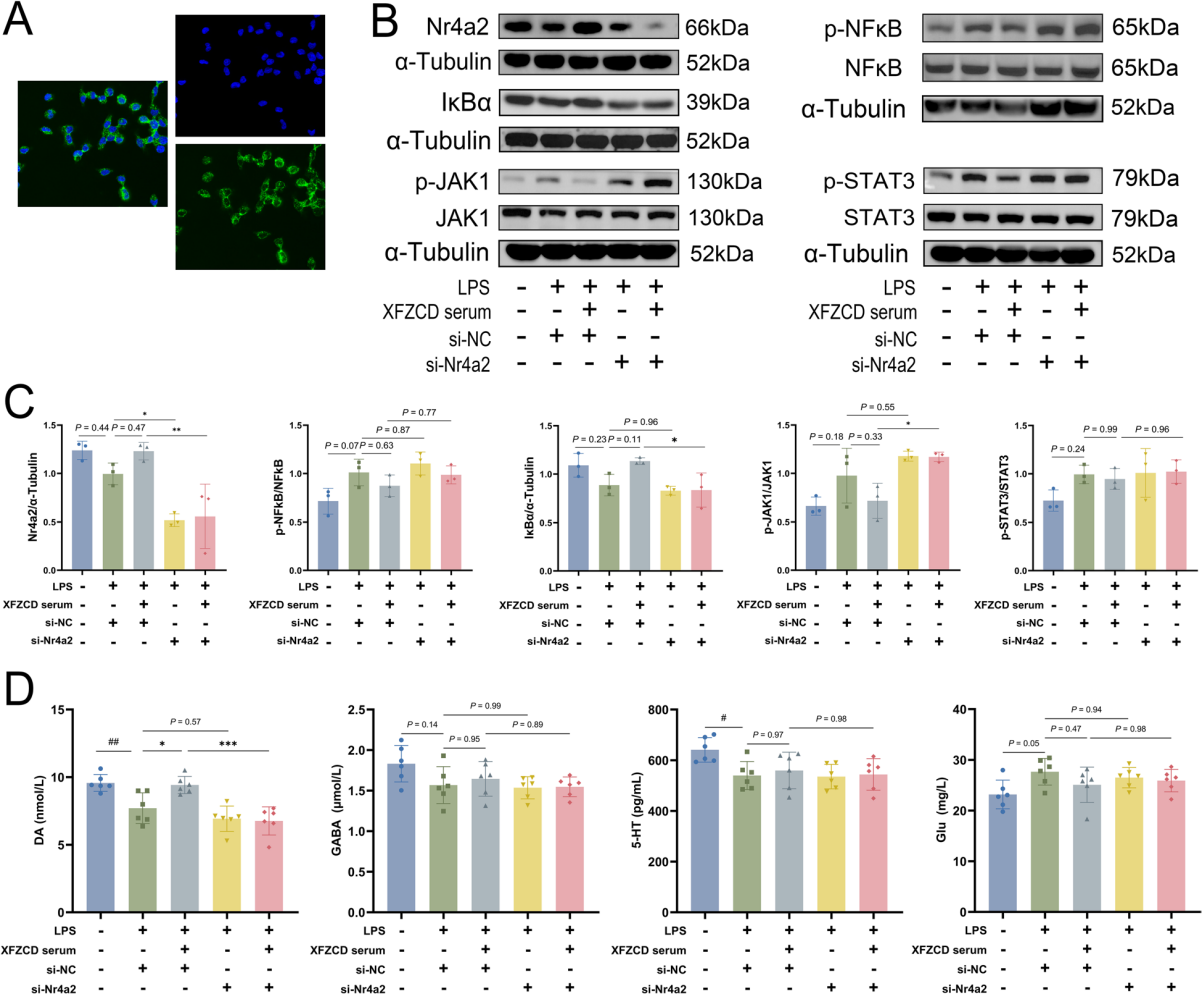
Nr4a2 knockdown attenuated the anti-inflammatory effect of XFZCD-containing serum in LPS-stimulated SH-SY5Y cells. **A** Transfection efficiency of FAM-labeled siRNA in SH-SY5Y cells; **B** WB bands of Nr4a2, IκBα, p-NFκB/NFκB, p-JAK1/JAK1, and p-STAT3/STAT3; **C** WB quantitative analysis of Nr4a2, IκBα, p-NFκB/NFκB, p-JAK1/JAK1, and p-STAT3/STAT3; **D** Effects of Nr4a2 knockdown on neurotransmitter levels including DA, 5-HT, GABA, and Glu were detected after LPS stimulation. Data are presented as mean ± SD. ^#^*P* < 0.05, ^##^ *P* < 0.01, ^###^*P* < 0.001 vs. CON group; *P < 0.05, **P < 0.01, ***P < 0.001 vs. MOD group; no symbol indicates no significant difference (*P* > 0.05)

The silencing of Nr4a2 via siRNA was conducted in SH-SY5Y cells challenged with LPS. At 24 h post-transfection, successful cellular transfection was validated through the observation of bright green fluorescence from FAM-labeled negative-control siRNA, alongside DAPI nuclear counterstaining (Fig. 6A). The efficiency of Nr4a2 knockdown was further confirmed via WB. Under si-NC conditions, treatment with XFZCD-containing serum effectively restored Nr4a2 expression and inhibited the excessive activation of NFκB/JAK-STAT induced by LPS (Fig. 6B-C). Conversely, these protective effects of XFZCD were significantly diminished following the depletion of Nr4a2. Compared to the LPS group, Nr4a2-knockdown (KD) cells demonstrated a marked downregulation of Nr4a2, along with reduced IκBα expression and increased phosphorylation of NFκB, JAK1, and STAT3. This evidence underscores the critical regulatory function of Nr4a2 in mitigating hyperactive pro-inflammatory signaling and highlights its central role in the anti-inflammatory effects of XFZCD.

As shown in Fig. 6D, the quantification of intracellular neurotransmitters demonstrated that LPS stimulation resulted in a significant depletion of DA (*P* < 0.01) and 5-HT (*P* < 0.05), as well as a trend towards reduced GABA (*P* = 0.14) and increased Glu (*P* = 0.05) compared to normal cells. In the presence of si-NC, the administration of XFZCD serum effectively ameliorated the neurotransmitter imbalances induced by LPS, exerting significant regulatory effects on the upregulation of DA (*P* < 0.05, vs LPS group). However, following the knockdown of Nr4a2, the beneficial modulatory effects of XFZCD on DA and Glu were nullified, and the modest improvements in 5-HT and GABA levels observed with XFZCD treatment were also diminished. These findings underscore the critical regulatory role of Nr4a2 in mediating the effects of XFZCD on neurotransmitter homeostasis and emphasize its essential importance in restoring DA balance.

## Discussion

### XFZCD exerts comprehensive therapeutic efficacy against TD

The pathological mechanisms underlying TD are characterized by considerable complexity. Emerging evidence has highlighted critical roles of neuroinflammation [17–19] and oxidative stress [20], and neurotransmitter imbalance [3, 19] in the progression of TD. In this study, multi-dimensional analyses demonstrated that XFZCD exerts comprehensive therapeutic effects by targeting both central and peripheral pathways. Network pharmacology research based on compounds indicates that XFZCD can engage in dopaminergic synapses, JAK-STAT signaling, and other neurotransmitter or inflammation-related pathways by targeting multiple key genes, such as AKT1, TNF, STAT3. Functionally, XFZCD inhibited microglial activation in the striatum, alleviated central neuroinflammation, reduced excessive oxidative stress, and restored the balance of DA, 5-HT, GABA, and glutamate, thereby ameliorating pathological damage in TD rats. Mechanistically, both rat experiments and Nr4a2-silencing experiments confirmed that XFZCD exerts anti-inflammatory and neurotransmitter-regulatory effects in TD.

Beyond central regulation, 16S rRNA and metabolomics analyses revealed that XFZCD remodels gut microbiota by restoring the *Firmicutes/Bacteroidetes* ratio and enriching beneficial genera, while reversing TD-related serum bile acid disturbances. These changes exhibited a strong correlation with the levels of inflammatory factors and neurotransmitters, as determined by Spearman analyses. Taken together, multi-omics combined cellular validation systematically clarifies that XFZCD alleviates TD lesions via a dual central-peripheral regulatory mode. Benefiting from this multi-target regulatory characteristic alongside its safety, XFZCD is a reliable candidate drug for TD therapy, providing valuable directions for subsequent basic and clinical research.

### Nr4a2 is a novel target for the treatment of TD

The nuclear receptor subfamily 4 group A member 2 (Nr4a2), also known as nuclear receptor related 1 protein (Nurr1), is widely expressed in the central nervous system. It plays a crucial role in maintaining the functional integrity of dopaminergic neurons [21, 22] and modulates neuroinflammation [23, 24], thereby serving as a pivotal molecule mediating neural homeostasis. Given that both dopaminergic dysfunction and excessive neuroinflammation are central to the pathogenesis of TD, Nr4a2 may serve as a critical molecular nexus linking neurotransmitter imbalances and inflammatory damage in the progression of TD.

Nr4a2 facilitates the differentiation of dopaminergic precursors into functional neurons and regulates genes for DA synthesis and metabolism, enhancing DA production, synaptic transmission and neuron function [25, 26]. Overexpression of Nr4a2 in mice has been reported to mitigate neuronal loss, reduce microglial proliferation, and alleviate hippocampal neuronal damage. Nr4a2 also indirectly protects neurons by inhibiting the expression of inflammatory genes in microglia and astrocytes [27, 28]. Under physiological conditions, Nr4a2 suppresses the activity of NFκB-p65 and recruits the CoREST complex to clear NFκB-p65, effectively inhibiting the expression of pro-inflammatory factors such as IL-1β and TNFα. Conversely, reduced Nr4a2 leads to excessive activation of the NFκB and triggers the cascading activation of inflammatory signaling including JAK1/STAT3 and astrocyte activation [28, 29].

In alignment with existing literature, this study corroborated that TD rats demonstrated significantly downregulated expression of Nr4a2 and IκBα, alongside heightened activation of NFκB, JAK1, and STAT3, as well as elevated serum levels of the pro-inflammatory cytokines TNFα and IL-6. Treatment with XFZCD effectively reversed these pathological alterations and markedly restored DA levels in the brain of TD rats. Molecular docking analyses further demonstrated favorable binding affinities between the core compounds of XFZCD and Nr4a2. Importantly, the knockdown of Nr4a2 nullified the anti-inflammatory and DA-restoring effects of XFZCD in LPS-stimulated SH-SY5Y cells, thereby validating Nr4a2 as an indispensable mediator of the therapeutic actions observed.

These findings support the novel hypothesis that Nr4a2 serves as a critical target for the treatment of TD. Further investigation into the specific mechanisms by which Nr4a2 regulates neuroinflammation and dopaminergic neurons is warranted and presents a promising direction for future research.

### Gut-brain axis is a potential effective approach for the treatment of TD

Clinical studies have demonstrated that patients with TD often present with gut microbiota dysbiosis, a condition that is strongly correlated with the severity of their tic symptoms [30–32]. Accumulating studies has elucidated the involvement of the gut-brain axis in neurological disorders [33, 34], a concept that has gained significant attention in the study of TD pathogenesis [35]. The gut microbiota participates in neurodevelopment by synthesizing various metabolites and neurotransmitters, including DA, 5-HT, GABA, and secondary bile acids. Neurotransmitters derived from microbes can influence microglial activation, while secondary bile acids and amino acid derivatives may further modulate central inflammatory responses, partly through microglial mediation. Some studies have demonstrated that fecal microbiota transplantation (FMT) effectively ameliorates TD symptoms [36, 37].

In this study, 16S rRNA and untargeted metabolomic data demonstrated obvious gut microbiota disturbances and systemic bile acid disorders in IDPN-induced TD rats. The model rats exhibited a disrupted *Firmicutes/Bacteroidetes* ratio and decreased abundances of *Ligilactobacillus, Bifidobacterium,* and *Romboutsia*, along with reduced serum levels of TUDCA and abnormal accumulation of bile acid derivatives. Spearman correlation analysis confirmed significant associations between these beneficial genera and circulating bile acid profiles.

Functionally, *Ligilactobacillus* and *Bifidobacterium* are involved in the regulation of bile acids through bile salt hydrolase activity [38, 39], while *Romboutsia* plays a role in the metabolism of carbohydrate, amino acid, and vitamin metabolism to provide precursors for bile acid biosynthesis [40]. In alignment with clinical observations of reduced *Bifidobacterium* levels in children with TD [31], all three genera were significantly suppressed in the model rats. Nevertheless, treatment with XFZCD effectively restored their abundance, corrected disordered bile acid homeostasis, and favorably modulated the levels of DA, 5-HT, GABA, and Glu in the brain, thereby conferring a neuroprotective effect. Collectively, the restructuring of intestinal flora to normalize amino acid and bile acid metabolism, in conjunction with Nr4a2-mediated central suppression of neuroinflammation and neurotransmitter rebalancing, represents a promising therapeutic strategy for TD.

### XFZCD restores neurotransmitter homeostasis to treat TD

The imbalance of neurotransmitter level is widely recognized as a key pathogenic mechanism in TD, primarily involving abnormality of the cortico-striatal-thalamo-cortical (CSTC) neural circuit. DA, Glu and GABA are essential neurotransmitters that regulate CSTC circuit function and are significantly implicated in TD pathogenesis [41]. This study demonstrates that XFZCD enhances the expression of Nr4a2, which facilitates dopaminergic neuron function and directly increases DA production, thereby restoring neurotransmitter homeostasis through central genetic regulation [27, 42, 43]. This mechanism accounts for the observed restoration of DA levels and the subsequent reduction of motor tics in TD rat models. Cell experiments further supported the positive effect of XFZCD-containing serum on DA and other neurotransmitters, which is abolished in the Nr4a2 knockdown. these finding confirmed the direct protective effect of XFZCD on neurons and restores neurotransmitter homeostasis in an Nr4a2-dependent manner.

In addition, the regulation of XFZCD on gut microbiota also contributes to neurotransmitter regulation via amino acid metabolism and bile acid metabolism. Specifically, the intestinal microbiota influences amino acid metabolism to supply precursors for the synthesis of DA and other neurotransmitters, while also normalizing the bile acid profile to enhance the metabolic environment conducive to the absorption of precursors [33, 44]. Certain bile acids, such as TUDCA, are capable of crossing the blood–brain barrier and can mitigate the release of proinflammatory factors by inhibiting the overactivation of microglia, thereby alleviating the excitotoxicity associated with elevated Glu levels [45, 46]. In summary, XFZCD facilitates neurotransmitter rebalancing by central targeting Nr4a2 and peripheral modulating intestinal flora, thereby contributing to the treatment of TD.

### XFZCD suppresses neuroinflammation to treat TD

Recently, neuroinflammation has been proposed in the pathogenetic mechanism of TD, and the inhibition of neuroinflammation has been shown to effectively control the symptoms of TD [47]. This study demonstrates that XFZCD exerts anti-inflammatory effects in TD rats through Nr4a2-mediated central inflammation and intestinal flora-mediated peripheral inflammation.

XFZCD upregulates Nr4a2 in the brain, thereby inhibiting the NFκB and JAK-STAT pathways, which are critical pathways involved in neuroinflammation due to microglial activation [48]. This results in a reduction of pro-inflammatory cytokine release, including TNF-α and IL-6, and the suppression of microglial activation, thereby mitigating neuroinflammation [27, 28]. In our study, the siRNA-mediated knockdown of Nr4a2 provided reverse validation for this mechanism. After Nr4a2 was silenced, the inhibitory effects of XFZCD on NFκB/JAK-STAT activation and pro-inflammatory cytokine expression were weakened or reversed, indicating that Nr4a2 is a key upstream mediator required for XFZCD induced suppression of neuroinflammation.

Simultaneously, XFZCD inhibits *Bacteroidetes* and upregulates *Firmicutes*, which not only reshapes the intestinal microbial environment barrier [49, 50] and reduces peripheral inflammation but also diminishes neuroinflammation by regulating in vivo metabolism via the gut-brain axis [51–53]. This is evidenced by the restoration of the bile acid profile, notably the elevation of TUDCA levels. TUDCA, which can traverse the blood–brain barrier [54, 55], has exhibited anti-inflammatory properties in various models by inhibiting the STING and NFκB pathways [46, 56]. Consequently, the reversal of gut microbiota dysbiosis and the normalization of serum bile acid metabolism attenuate the activation of peripheral pro-inflammatory factors, thereby indirectly reducing central inflammation. In summary, XFZCD alleviates neuroinflammation by targeting Nr4a2 and modulating intestinal flora, representing a primary therapeutic strategy for treating TD.

The present study has systematically elucidated the coordinated mechanisms involving central Nr4a2 and peripheral gut microbiota in the action of XFZCD against TD, employing multi-omics approaches and cellular validation. Despite the comprehensive nature of these findings, several limitations persist within this study. Firstly, the study quantified total neurotransmitter levels in tissue homogenates rather than examining the dynamic release patterns and concentrations within the synaptic cleft. Future research employing in vivo microdialysis to measure extracellular striatal neurotransmitters is necessary to provide more direct evidence for characterizing synaptic transmission and local neural circuit activity. Secondly, although correlative changes in intestinal flora were observed following XFZCD treatment, a causal relationship between the altered gut microbiota and the anti-TD effects has not been established. Studies involving fecal microbiota transplantation (FMT) or antibiotic depletion experiments are required to determine whether XFZCD confers therapeutic benefits via the gut-brain axis. Thirdly, the mechanistic insights were derived from IDPN-induced rat models and in vitro cell assays; thus, further clinical trials are essential to evaluate the translational potential of XFZCD for the treatment of TD in humans.

## Conclusion

**Fig. 7:**
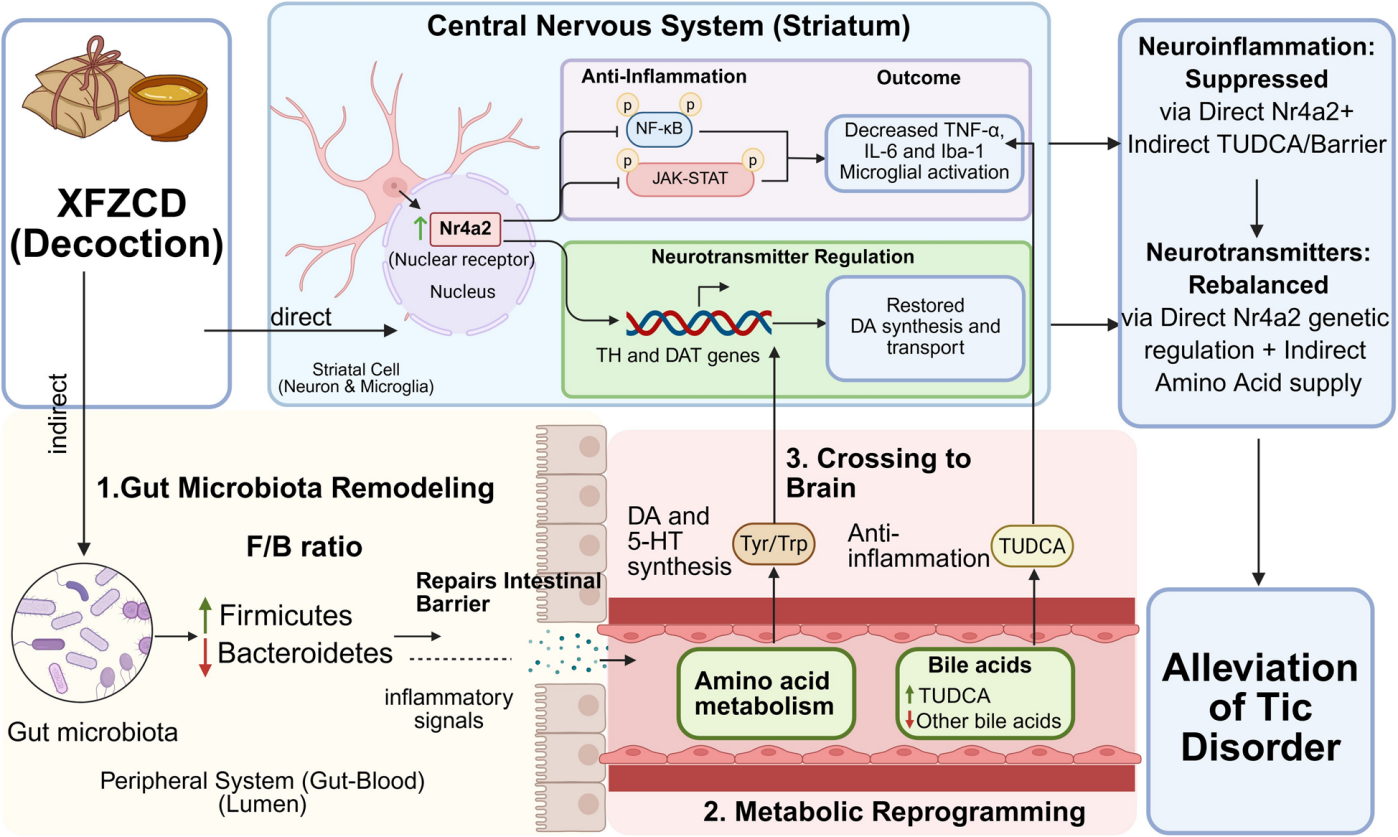
Mechanism diagram of XFZCD in treating TD

This study illustrates that XFZCD shows substantial therapeutic efficacy in mitigating the symptoms of TD. The underlying mechanism is intricately linked to the modulation of neuroinflammation and neurotransmitter equilibrium, co-regulated by Nr4a2 and the gut microbiota. Notably, the dual regulatory function of Nr4a2 in attenuating inflammation and enhancing dopaminergic activity highlights its potential as a promising therapeutic target for managing TD (Fig. 7).

## Supplementary Information

Supplementary material 1.

Supplementary material 2.

Supplementary material 3.

## Data Availability

No datasets were generated or analysed during the current study.

## Abbreviations

5-HT: Serotonin
BPC: Base peak chromatogram
DA: Dopamine
DEGs: Differentially expressed genes
ELISA: Enzyme-linked immunosorbent assay
FC: Fold change
FPKM: Fragments Per Kilobase of exon model per Million mapped fragments
GABA: Gamma-aminobutyric acid
Glu: Glutamic acid
GO: Gene Ontology
GPx: Glutathione peroxidase
IBA1: Ionized calcium-binding adapter molecule 1
IDPN: 3,3-Iminodipropionitrile
IL-6: Interleukin-6
KEGG: Kyoto Encyclopedia of Genes and Genomes
LPS: Lipopolysaccharides
MDA: Malondialdehyde
MS: Mass spectrometry
OFT: Open field test
PCA: Principal component analysis
PPI: Protein–protein interaction networks
SOD: Superoxide dismutase
TCM: Traditional Chinese medicine
TNFα: Tumor necrosis factor α
TD: Tic disorder
UPLC-MS: Ultra Performance Liquid Chromatography-Tandem Mass Spectrometry
VIP: Variable Importance in the Projection
WB: Western Blot
XFZCD: *Xifeng Zhichou* decoction
YGTSS: Yale Global Tic Severity Scale
