# Supplementary material for: Xifeng Zhichou decoction mitigates tic disorder on juvenile rats by regulating neuroinflammation and neurotransmitter homeostasis: dual modulation of Nr4a2 and gut microbiota
Source: Chin Med. 2026 Jul 8;21:183. doi: 10.1186/s13020-026-01464-3 (PMC13343772; doi:10.1186/s13020-026-01464-3)
Supplement: Supplementary file 1 — Supplementary material 1. [file 13020_2026_1464_MOESM1_ESM.docx]

| **Table S1 Compounds of XFZCD (* means both the positive ion mode and the negative ion mode can be determined)** | | | | | | | | |
| --- | --- | --- | --- | --- | --- | --- | --- | --- |
| **Num.** | **Rt** | **m/z** | **Ion** | **Compounds** | **Molecular Formula** | **Fragment Ions** | **Source** | **Compound ID in network pharmacology analysis** |
| 1 | 0.7 | 191.056 | M-H | Citric Acid | C_6_H_8_O_7_ | 127.0357,173.0389,191.0496 | Tianma, Mugua, Jiangcan | A6 |
| 2 | 0.71 | 171.0294 | M-H | Decanoic acid | C_10_H_20_O_2_ | 81.0313,109.0256,127.0355 | Jiangcan | JC1 |
| 3 | 0.74 | 665.2138 | M-H | Stachyose | C_24_H_42_O_21_ | 443.1205,485.1286,665.1844 | Dihuang | SDH11 |
| 4 | 0.76 | 341.1077 | M-H | Sucrose | C_12_H_22_O_11_ | 143.0289,161.0390,179.0486,341.0935 | Tianma, Baishao | A1 |
| 5 | 0.83 | 381.0786 | M+H | Tetrahydrodemethoxycurcumin | C_20_H_22_O_5_ | 201.0159,219.0254 | Yujin | YJ1 |
| 6 | 0.97 | 173.0456 | M-H | arginine | C_6_H_14_N_4_O_2_ | 111.0411,131.0783,173.0383 | Jiangcan, Muli | A2 |
| 7 | 1.1 | 375.1296 | M-H | Octahydrocurcumin | C_21_H_28_O_6_ | 195.0598,345.1069,375.1154 | Yujin | YJ5 |
| 8 | 1.29 | 542.2072 | M+NH_4_ | Rehmannioside A | C_21_H_32_O_15_ | 309.0957,327.1078,345.1182 | Dihuang | SDH1 |
| 9^*^ | 1.44 | 394.1701 | M+NH_4_ | 8-Epiloganic acid | C_16_H_24_O_10_ | 151.0750,161.0595,179.0701 | Dihuang | SDH2 |
| 10 | 1.44 | 478.1539 | M+NH_4_ | Parishin E | C_29_H_24_O_13_ | 107.0481 | Tianma | TM1 |
| 11 | 1.61 | 183.1023 | M-H | Methyl gallate | C_8_H_8_O_5_ | 139.1194,183.0929 | Baishao | BS5 |
| 12 | 1.67 | 542.2057 | M+NH_4_ | Rehmannioside B | C_21_H_32_O_15_ | 201.0757,267.0854,327.1066,345.1168 | Dihuang | SDH3 |
| 13 | 1.75 | 380.1546 | M+NH_4_ | Catalpol | C_15_H_22_O_10_ | 165.0543,183.0649 | Dihuang | SDH5 |
| 14 | 1.78 | 380.1542 | M+NH_4_ | Monomelittoside | C_15_H_22_O_10_ | 163.0596,165.0543,183.0649 | Dihuang | SDH4 |
| 15 | 2.02 | 353.088 | M-H | Chlorogenic Acid | C_16_H_18_O_9_ | 85.0255,191.0479 | Gouteng, Mugua | A3 |
| 16 | 2.22 | 253.1274 | M+H | Methyl3,4,5-Trimethoxycinnamate | C_13_H_16_O_5_ | 109.0270,145.0478,163.0581 | Yuanzhi | YZ1 |
| 17 | 2.65 | 137.0543 | M-H | 4-Hydroxybenzoic acid | C_7_H_6_O_3_ | 75.0201,93.0311, | Tianma, Mugua | A4 |
| 18 | 2.88 | 241.1192 | M-H | 7-O-ethyl guaiacylglycerol | C_12_H_18_O_5_ | 141.0977,162.8874,195.1041,197.1219,241.1084 | Dihuang | SDH10 |
| 19 | 3.31 | 378.1749 | M+NH_4_ | 1-O-β-D-glucopyranosylpaeonisuffron | C_16_H_24_O_9_ | 163.0746;181.0851;199.0957 | Baishao | BS9 |
| 20 | 3.46 | 493.1187 | M-H | Polygalaxanthone VI | C_23_H_26_O_12_ | 283.0351,313.0437,493.1018 | Yuanzhi | YZ12 |
| 21^*^ | 4.23 | 412.1177 | M-H | L-γ-Glutamyl-S-[(4-hydroxyphenyl) methyl]-L-cysteinylglycine | C_17_H_23_N_3_O_7_S | 288.0524,306.0638,412.0989 | Tianma | TM8 |
| 22 | 4.32 | 203.0818 | M-H | tryptophan | C_11_H_12_N_2_O_2_ | 142.0601,2030781 | Jiangcan, Muli | A5 |
| 23 | 4.39 | 380.154 | M+NH_4_ | (5Z)-7-Oxozeaenol | C_19_H_22_O_7_ | 191.0599,309.0952,327.1067 | Yujin | - |
| 24^*^ | 4.45 | 685.2205 | M-H | Rehmannioside D | C_27_H_42_O_20_ | 183.0643,325.1117,327.1062,489.1569 | Dihuang | SDH7 |
| 25 | 4.46 | 415.1795 | M+H | aeginetic acid 5-O-β-D-quinovoside | C_21_H_34_O_8_ | 145.0492,163.0596,253.1282 | Dihuang | - |
| 26^*^ | 4.53 | 523.166 | M-H | Melittoside | C_21_H_32_O_15_ | 331.0714,361.0959,463.1274,523.1420 | Dihuang | SDH6 |
| 27 | 4.59 | 353.0877 | M-H | Desmethylicaritin | C_20_H_18_O_6_ | 173.0398,179.0287,191.0493,353.0738 | Shichangpu | SCP1 |
| 28^*^ | 4.71 | 347.1349 | M-H | Ajugol | C_15_H_24_O_9_ | 167.0653,185.0733,347.1080 | Dihuang | SDH8 |
| 29^*^ | 4.83 | 727.2125 | M-H | Parishin B | C_32_H_40_O_19_ | 269.1019,299.0763,405.1180 | Tianma | TM3 |
| 30 | 4.88 | 304.1637 | M+NH_4_ | Gastrodin | C_13_H_18_O_7_ | 107.0491,261.1800 | Tianma | TM2 |
| 31^*^ | 4.92 | 607.1899 | M-H | Polygalin B | C_28_H_32_O_15_ | 163.0594,267.0854,325.1135 | Yuanzhi | YZ2 |
| 32 | 4.97 | 461.1303 | M-H | Decaffeoylverbascoside | C_20_H_30_O_12_ | 281.0547,299.0643,461.1117 | Dihuang | SDH9 |
| 33 | 5 | 301.0911 | M+H | Rhamnocitrin | C_16_H_12_O_6_ | 139.0391,265.0706,283.0785 | Dihuang | - |
| 34 | 5.04 | 371.0773 | M-H | Veraguensin | C_22_H_28_O_5_ | 99.0053,121.0246,371.0853 | Shichangpu | SCP2 |
| 35 | 5.09 | 345.1541 | M+H | Theogallin | C_14_H_16_O_10_ | 137.0953,147.0797,165.0907,183.1017 | Baishao | BS7 |
| 36^*^ | 5.21 | 495.1513 | M-H | oxypaeoniflorin | C_23_H_28_O_12_ | 151.0745,161.0591,179.0699,197.0804 | Baishao | BS6 |
| 37 | 5.31 | 517.1585 | M-H | Arillatose B | C_22_H_30_O_14_ | 175.0340,193.0431,517.1329 | Yuanzhi | YZ5 |
| 38 | 5.4 | 369.1171 | M+H | Curcumin | C_21_H_20_O_6_ | 147.0433,175.0386,207.0651 | Yujin | YJ2 |
| 39^*^ | 5.45 | 429.1397 | M-H | Polygalatenoside A | C_19_H_26_O_11_ | 163.0550,307.0902,429.1950 | Yuanzhi | YZ3 |
| 40 | 5.83 | 385.1874 | M-H | (E)-7-Hydroxy-1,7-bis-(4-hydroxy-3-methoxyphenyl)-1-heptene-3,5-dione | C_21_H_22_O_7_ | 205.1163,205.1317,223.1225,385.1702 | Yujin | - |
| 41 | 5.94 | 464.1747 | M+NH_4_ | sacranoside A | C_21_H_34_O_10_ | 277.0929,285.0945,295.1024 | Baishao | BS8 |
| 42^*^ | 6.28 | 479.1566 | M-H | Paeoniflorin | C_23_H_28_O_11_ | 179.0697,197.0804,301.1065,319.1172 | Baishao | BS1 |
| 43^*^ | 6.65 | 498.1954 | M+NH_4_ | albiflorin | C_23_H_28_O_11_ | 327.0959,449.1259,479.1362 | Baishao | BS2 |
| 44 | 7.66 | 383.132 | M+Na | Dihydrocurcumin | C_21_H_22_O_6_ | 163.0381,191.0337;193.0852;221.0805 | Yujin | YJ3 |
| 45 | 7.66 | 561.1831 | M-H | glomeratose A | C_24_H_34_O_15_ | 133.0611,237.0679,323.0839,561.1542 | Yuanzhi | YZ6 |
| 46 | 8.42 | 389.2187 | M-H | 3,4-Dihydroverbenalin | C_17_H_26_O_10_ | 161.0385,179.0494,389.1969,389.2309 | Dihuang | SDH12 |
| 47^*^ | 9.26 | 995.3092 | M-H | Parishin A | C_45_H_56_O_25_ | 431.1451,461.1278,535.1789,567.1696 | Tianma | TM6 |
| 48 | 9.54 | 667.1901 | M-H | Tenuifoliside B | C_30_H_36_O_17_ | 367.0888,443.1071,461.1139,667.1634 | Yuanzhi | YZ7 |
| 49 | 10.12 | 679.3719 | M-H | Arillatose C | C_28_H_40_O_19_ | 445.2987,649.3313,679.3459 | Yuanzhi | YZ8 |
| 50 | 11.22 | 832.3204 | M+NH_4_ | Jionoside B2 | C_37_H_50_O_20_ | 145.0276,177.0540,485.1634,507.1838 | Dihuang | SDH13 |
| 51 | 11.56 | 679.5093 | M+H | Sibiricasaponin A | C_36_H_54_O_12_ | 209.1644,661.4988,679.5089 | Yuanzhi | YZ9 |
| 52 | 11.88 | 753.2287 | M-H | 3′,6-Disinapoylsucrose | C_34_H_42_O_19_ | 265.0610,529.1365,547.1459,753.1970 | Yuanzhi | YZ10 |
| 53 | 12.8 | 1103.489 | M-H | Watterose H | C_51_H_60_O_27_ | 1103.4892 | Yuanzhi | YZ11 |
| 54 | 13.08 | 1249.592 | M-H | Dalmaisiose D | C_57_H_70_O_31_ | 1249.5398 | Yuanzhi | - |
| 55 | 13.36 | 767.2172 | M-H | Tenuifoliside C | C_35_H_44_O_19_ | 349.0797,367.0898,529.1391,767.2172 | Yuanzhi | YZ13 |
| 56 | 13.62 | 1307.399 | M-H | Tenuifoliose J | C_59_H_72_O_33_ | 1101.2865,1119.2967 | Yuanzhi | - |
| 57 | 13.72 | 1326.426 | M+NH_4_ | Tenuifoliose I | C_59_H_72_O_33_ | 499.3061,1245.202,1245.3093 | Yuanzhi | YZ4 |
| 58 | 13.73 | 1337.411 | M-H | TenuifolioseD | C_60_H_74_O_34_ | 997.2710,1101.297,1119.3027,1161.3156 | Yuanzhi | - |
| 59 | 14.25 | 1349.409 | M-H | Tenuifoliose H | C_61_H_74_O_34_ | 1161.3125,1203.3256 | Yuanzhi | - |
| 60^*^ | 14.33 | 602.2226 | M+NH_4_ | benzoyl paeoniflorin | C_30_H_32_O_12_ | 249.0760,267.0863,301.1082,445.1485 | Baishao | BS3 |
| 61 | 15.14 | 263.1294 | M-H | Curcumenolactone C | C_15_H_20_O_4_ | 203.1013,219.1299,245.1074,263.1190 | Yujin | YJ6 |
| 62 | 16.1 | 1587.6997 | M-H | Onjisaponin F | C_75_H_112_O_36_ | 895.3405,1269.5534 | Yuanzhi | - |
| 63 | 16.34 | 277.1808 | M-H | Alpha-linolenic acid | C_18_H_30_O_2_ | 191.0740,233.1820,277.1700 | Jiangcan | JC4 |
| 64 | 16.87 | 383.1958 | M+H | Corynoxeine | C_22_H_26_N_2_O_4_ | 224.1274,351.1693,383.1956 | Gouteng | GT1 |
| 65 | 17.62 | 235.169 | M+H | Curmadione | C_15_H_22_O_2_ | 189.1285,189.1631,235.1684 | Yujin | YJ4 |
| 66 | 27.61 | 283.2645 | M-H | Parietin | C_16_H_12_O_5_ | 283.253 | Shichangpu | SCP3 |
