# Supplementary material for: Xifeng Zhichou decoction mitigates tic disorder on juvenile rats by regulating neuroinflammation and neurotransmitter homeostasis: dual modulation of Nr4a2 and gut microbiota
Source: Chin Med. 2026 Jul 8;21:183. doi: 10.1186/s13020-026-01464-3 (PMC13343772; doi:10.1186/s13020-026-01464-3)
Supplement: Supplementary file 2 — Supplementary material 2. [file 13020_2026_1464_MOESM2_ESM.docx]

| **Supplementary Table S2. Top 20 core compounds of XFZCD ranked by degree values in the herb-component-target network** | | | | |
| --- | --- | --- | --- | --- |
| **Num.** | **Compounds** | **Source** | **Degree** | **Binding affinities with Nr4a2** |
| 1 | Chlorogenic Acid | Gouteng Mugua | 18 | -7 |
| 2 | 4-Hydroxybenzoic acid | Tianma Mugua | 17 | -5.8 |
| 3 | Tryptophan | Jiangcan Muli | 17 | -6.5 |
| 4 | Sucrose | Baishao Tianma | 16 | -5.6 |
| 5 | Octahydrocurcumin | Yujin | 16 | -5.8 |
| 6 | Polygalin B | Yuanzhi | 16 | -6.8 |
| 7 | L-γ-Glutamyl-S-[(4-hydroxyphenyl) methyl]-L-cysteinylglycine | Tianma | 16 | -7.1 |
| 8 | Desmethylicaritin | Shichangpu | 16 | -7.7 |
| 9 | Veraguensin | Shichangpu | 16 | -6 |
| 10 | Decaffeoylverbascoside | Dihuang | 16 | -7.6 |
| 11 | Albiflorin | Baishao | 16 | -7.9 |
| 12 | Sibiricasaponin A | Yuanzhi | 16 | -7.5 |
| 13 | Dihydrocurcumin | Yujin | 16 | -6 |
| 14 | Paeoniflorin | Baishao | 16 | -8 |
| 15 | Corynoxeine | Gouteng | 16 | -6.2 |
| 16 | Gastrodin | Tianma | 16 | -6.8 |
| 17 | Curcumin | Yujin | 16 | -6.3 |
| 18 | Catalpol | Dihuang | 16 | -5.7 |
| 19 | Parishin A | Tianma | 16 | -7.9 |
| 20 | Dihydrocurcumin | Yujin | 16 | -6 |
