# Supplementary material for: Xifeng Zhichou decoction mitigates tic disorder on juvenile rats by regulating neuroinflammation and neurotransmitter homeostasis: dual modulation of Nr4a2 and gut microbiota
Source: Chin Med. 2026 Jul 8;21:183. doi: 10.1186/s13020-026-01464-3 (PMC13343772; doi:10.1186/s13020-026-01464-3)
Supplement: Supplementary file 3 — Supplementary material 3. [file 13020_2026_1464_MOESM3_ESM.pdf]

Striatum

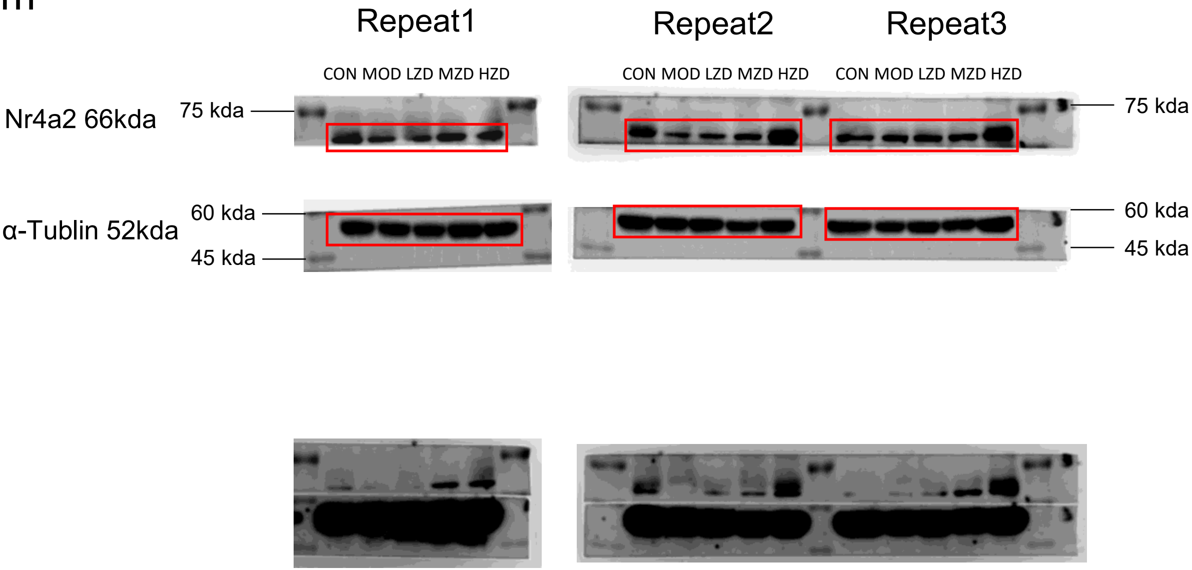

Repeat1
Repeat2
Repeat3
CON MOD LZD MZD HZD
CON MOD LZD MZD HZD
CON MOD LZD MZD HZD
Nr4a2 66kda
75 kda
75 kda
α-Tublin 52kda
60 kda
45 kda
60 kda
45 kda

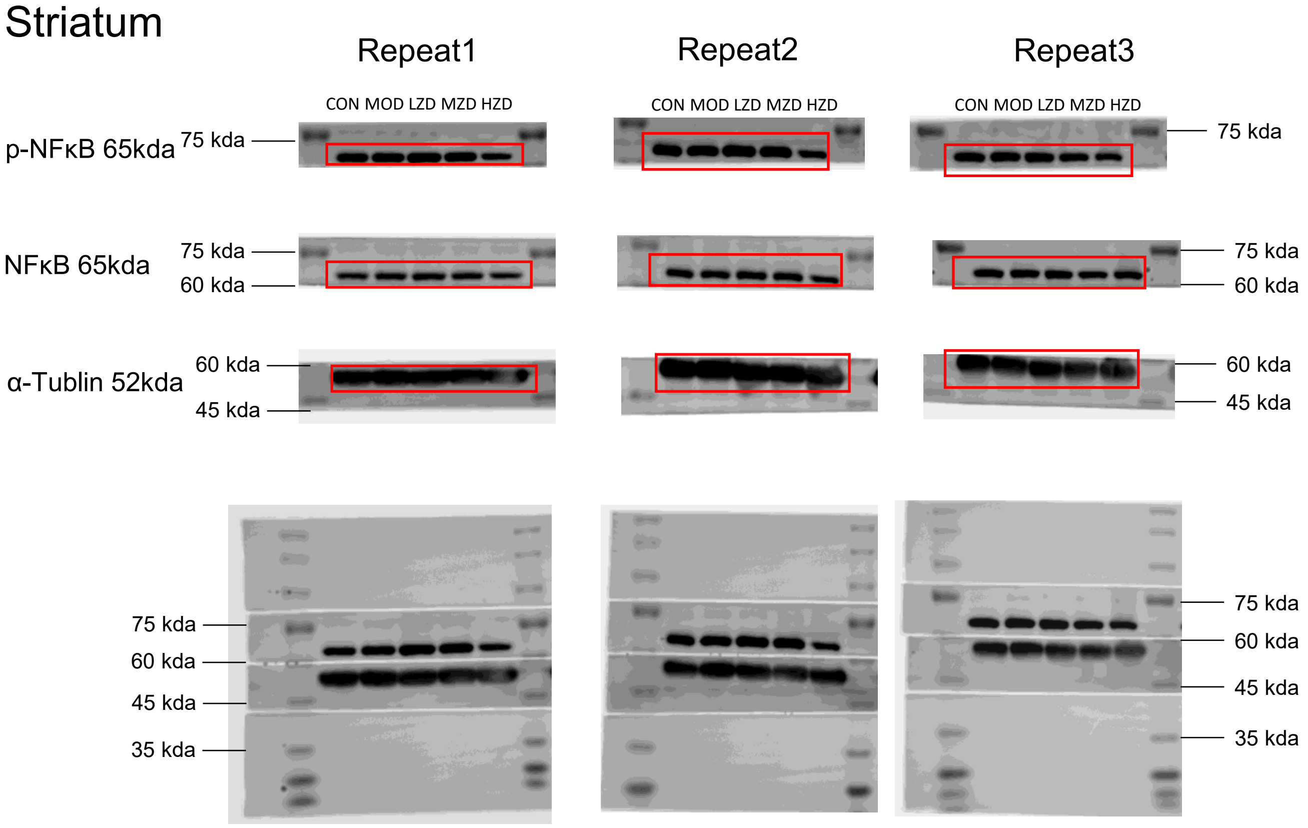
Striatum
Repeat1
Repeat2
Repeat3
CON MOD LZD MZD HZD
CON MOD LZD MZD HZD
CON MOD LZD MZD HZD
p-NFκB 65kda
NFκB 65kda
α-Tublin 52kda
75 kda
60 kda
45 kda
35 kda

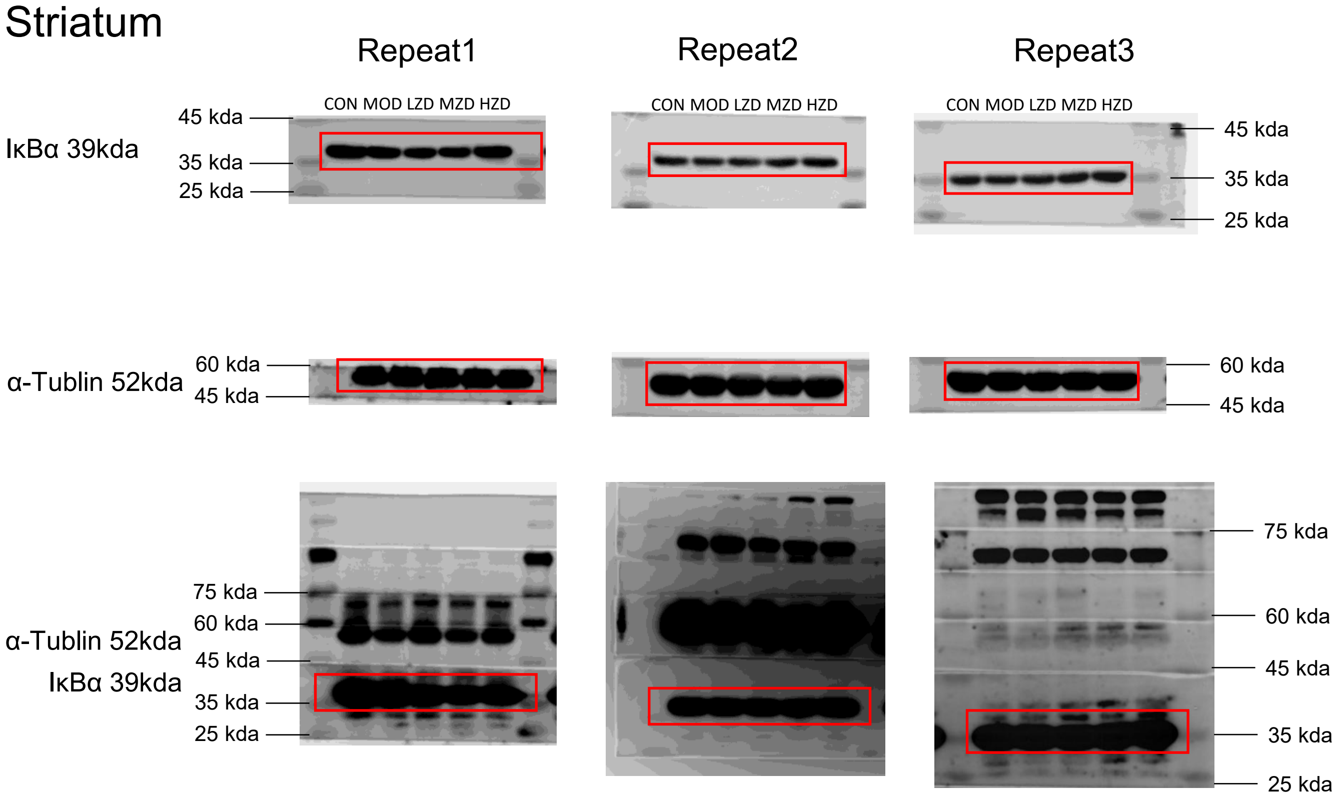
Striatum
Repeat1
Repeat2
Repeat3
CON MOD LZD MZD HZD
IκBα 39kda
45 kda
35 kda
25 kda
α-Tublin 52kda
60 kda
45 kda
75 kda
IκBα 39kda

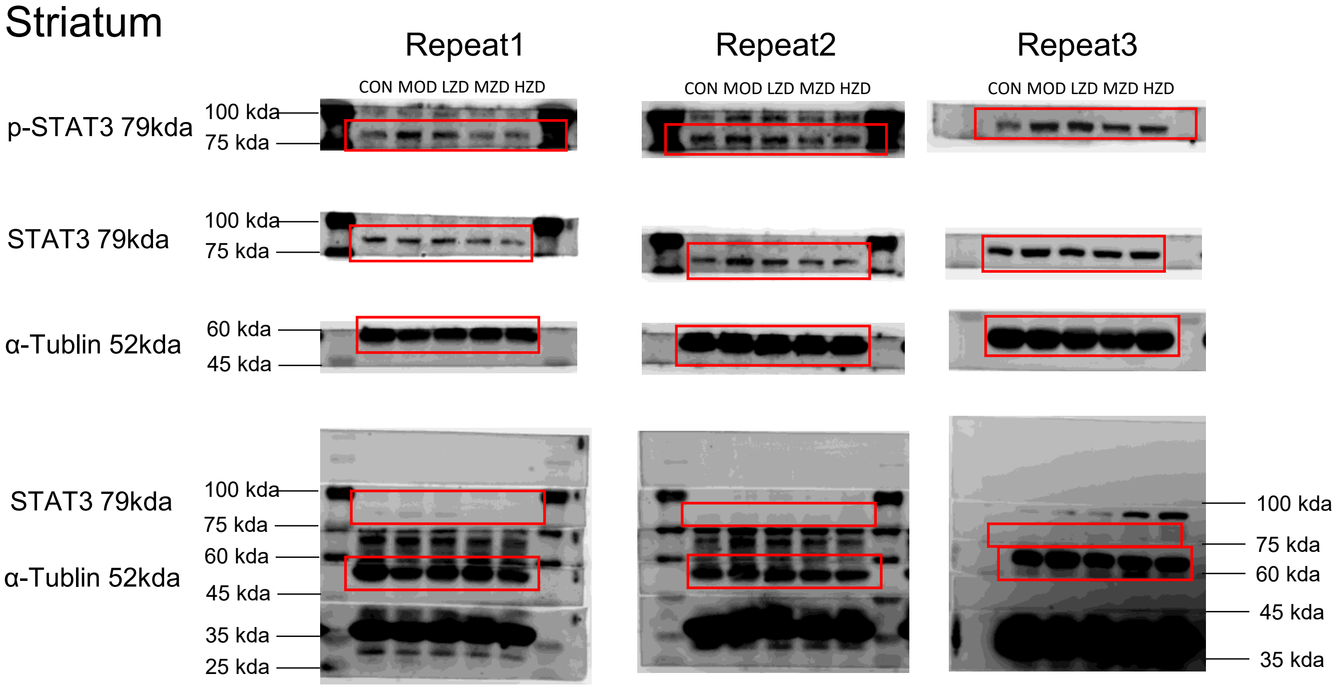
Striatum
Repeat1
Repeat2
Repeat3
CON MOD LZD MZD HZD
CON MOD LZD MZD HZD
CON MOD LZD MZD HZD
p-STAT3 79kda
100 kda
75 kda
STAT3 79kda
100 kda
75 kda
α-Tublin 52kda
60 kda
45 kda
STAT3 79kda
α-Tublin 52kda
100 kda
75 kda
60 kda
45 kda
35 kda
25 kda
100 kda
75 kda
60 kda
45 kda
35 kda

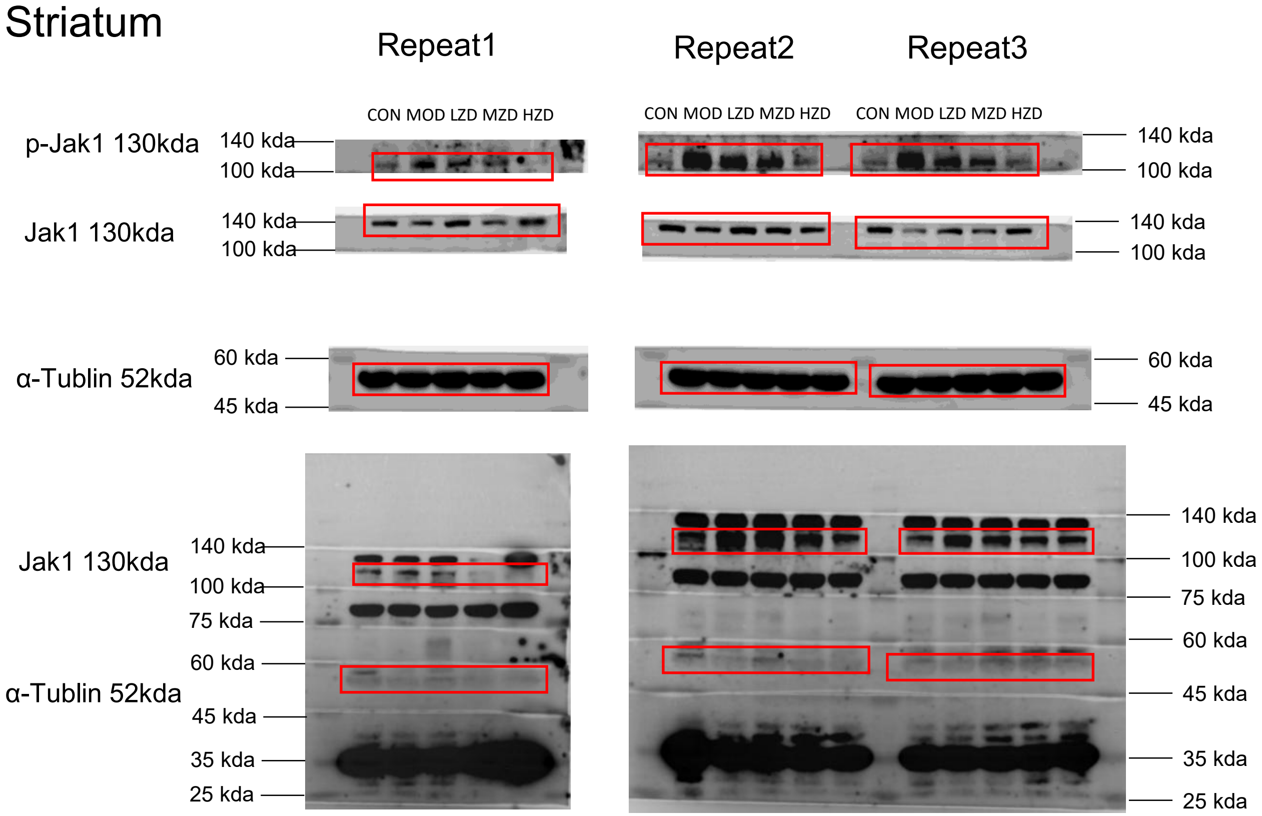
Striatum
Repeat1
Repeat2
Repeat3
CON MOD LZD MZD HZD
CON MOD LZD MZD HZD
CON MOD LZD MZD HZD
p-Jak1 130kda
140 kda
100 kda
Jak1 130kda
140 kda
100 kda
α-Tublin 52kda
60 kda
45 kda
Jak1 130kda
α-Tublin 52kda
140 kda
100 kda
75 kda
60 kda
45 kda
35 kda
25 kda

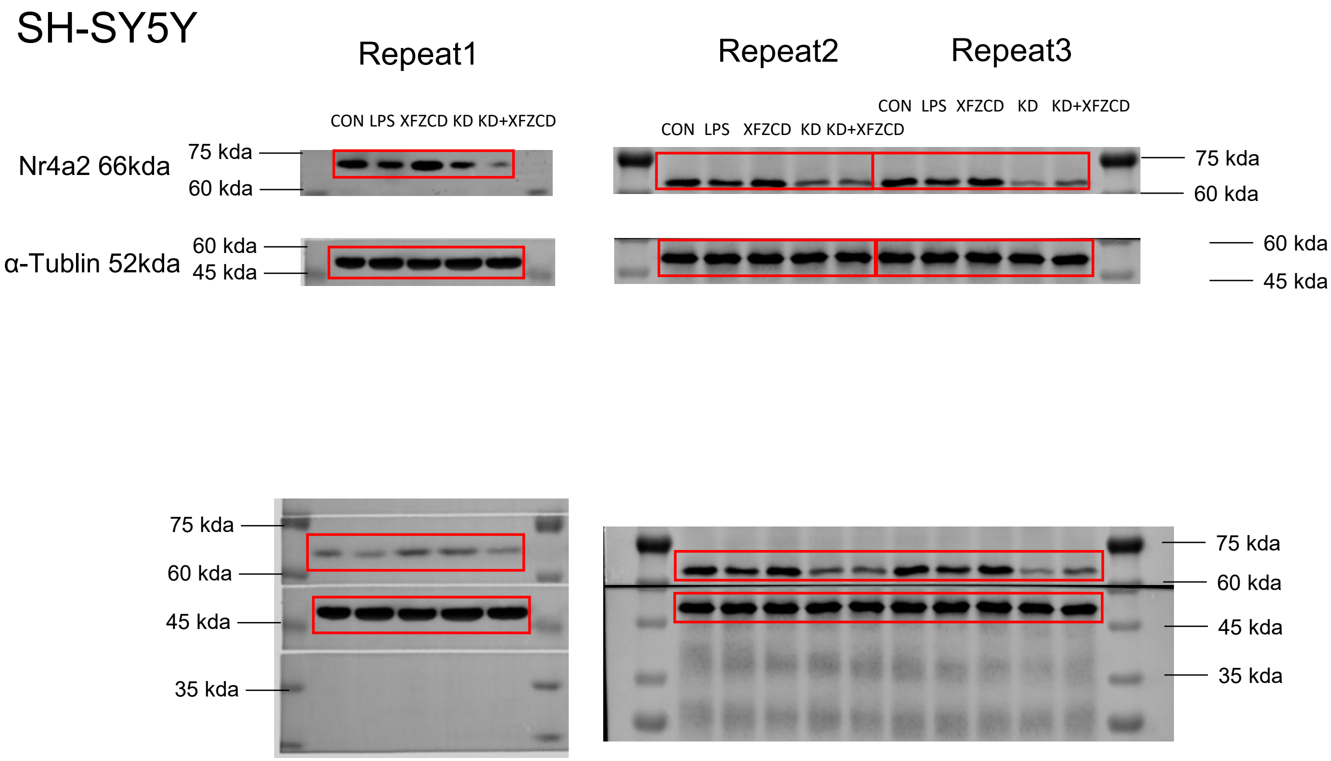

SH-SY5Y
Repeat1
Repeat2
Repeat3
CON LPS XFZCD KD KD+XFZCD
CON LPS XFZCD KD KD+XFZCD
CON LPS XFZCD KD KD+XFZCD
Nr4a2 66kda
α-Tublin 52kda
75 kda
60 kda
45 kda
35 kda

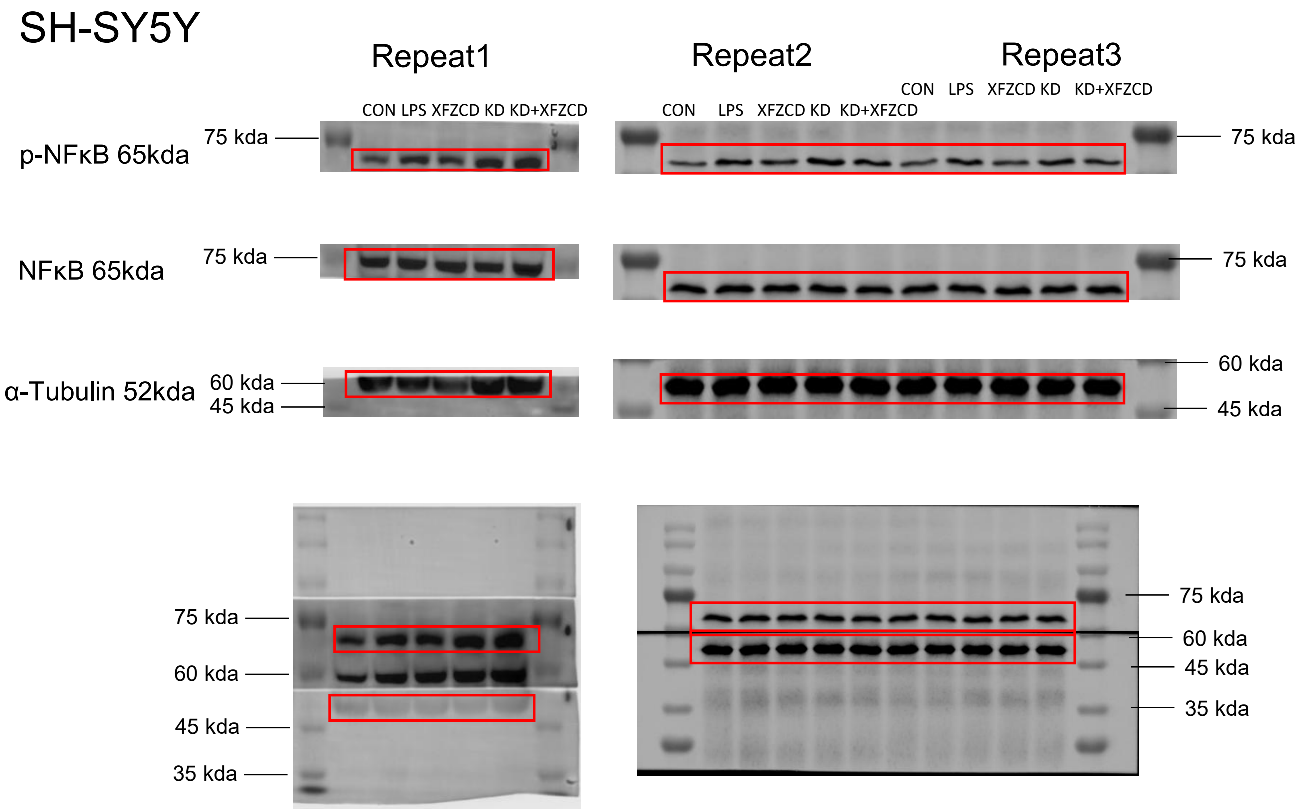

SH-SY5Y
Repeat1
Repeat2
Repeat3
CON LPS XFZCD KD KD+XFZCD
CON LPS XFZCD KD KD+XFZCD
CON LPS XFZCD KD KD+XFZCD
p-NFκB 65kda
NFκB 65kda
α-Tubulin 52kda
75 kda
60 kda
45 kda
35 kda

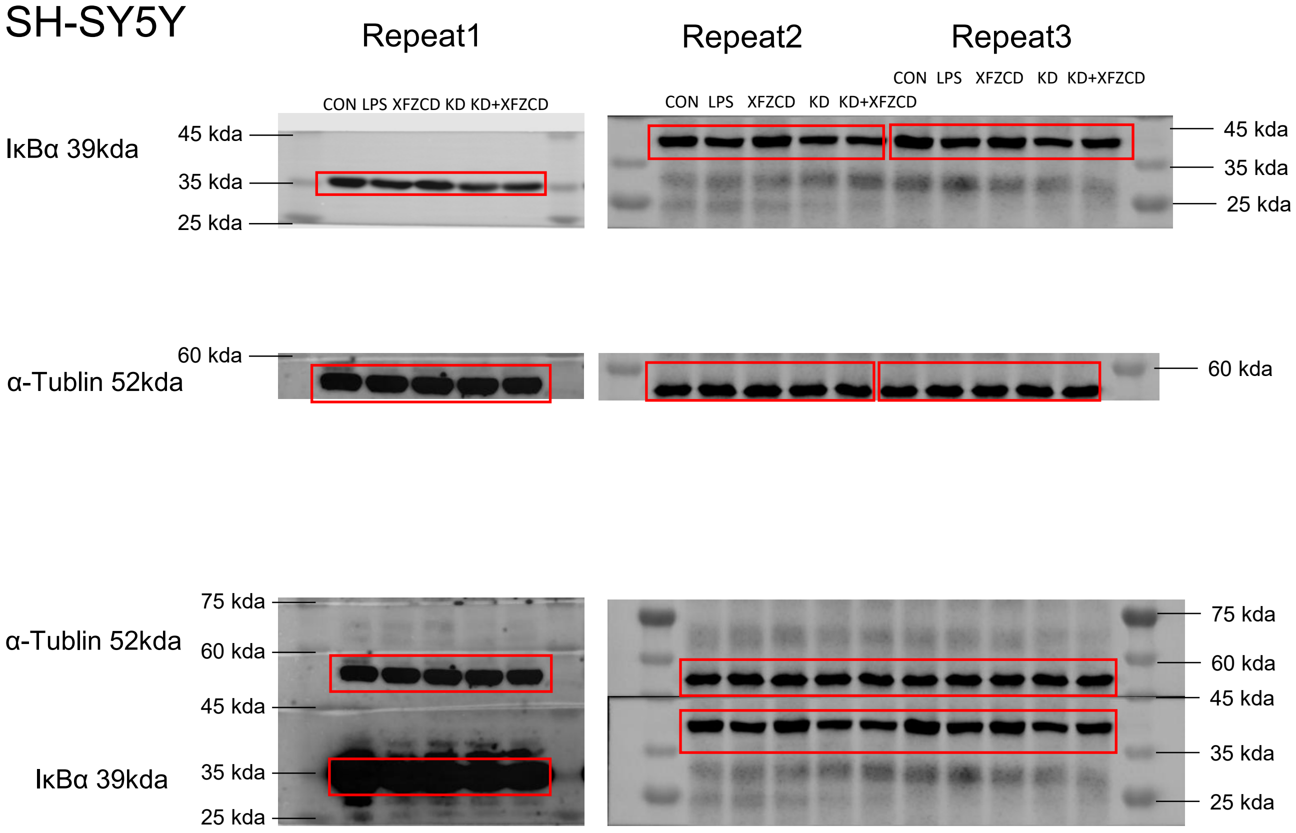

SH-SY5Y
Repeat1
Repeat2
Repeat3
CON LPS XFZCD KD KD+XFZCD
CON LPS XFZCD KD KD+XFZCD
CON LPS XFZCD KD KD+XFZCD
IκBα 39kda
45 kda
35 kda
25 kda
45 kda
35 kda
25 kda
α-Tublin 52kda
60 kda
60 kda
α-Tublin 52kda
IκBα 39kda
75 kda
60 kda
45 kda
35 kda
25 kda
75 kda
60 kda
45 kda
35 kda
25 kda

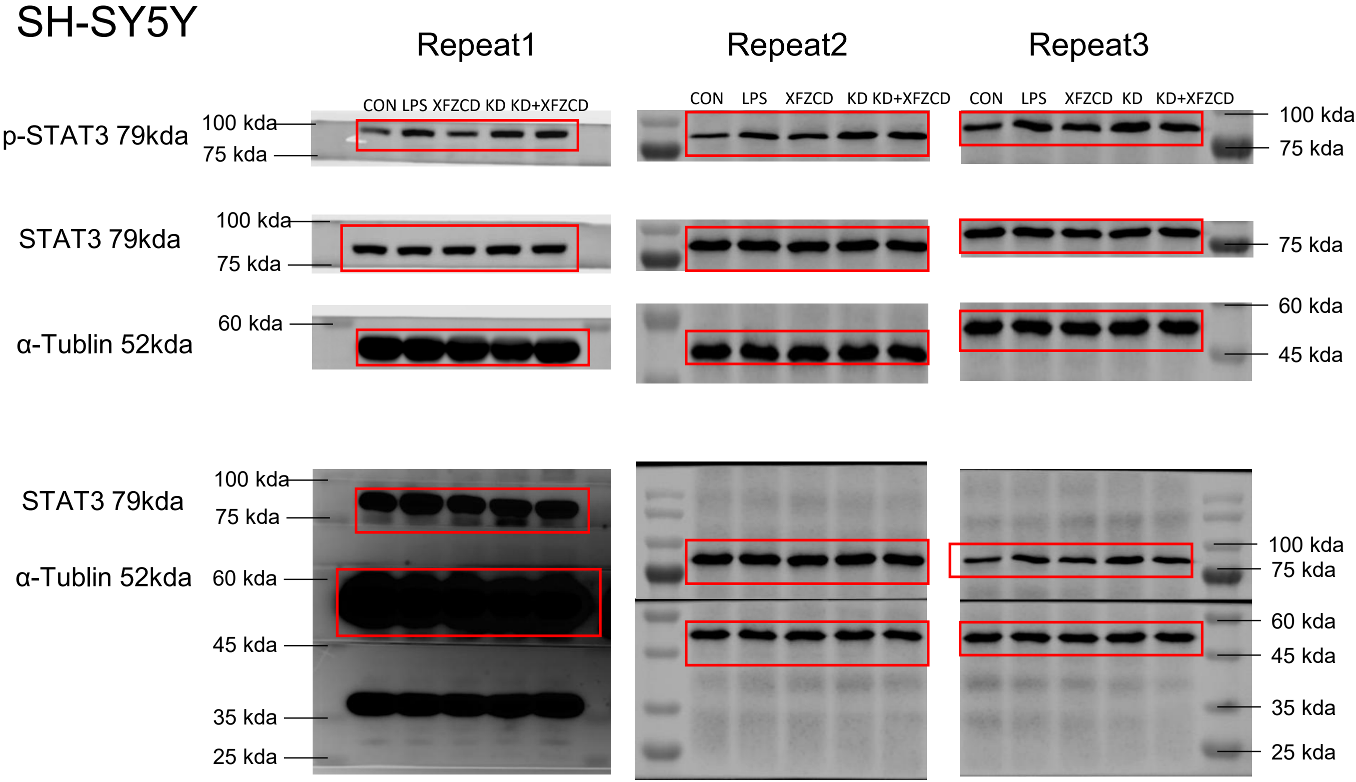
SH-SY5Y
Repeat1
Repeat2
Repeat3
CON LPS XFZCD KD KD+XFZCD
p-STAT3 79kda
STAT3 79kda
α-Tublin 52kda
STAT3 79kda
α-Tublin 52kda
100 kda
75 kda
60 kda
45 kda
35 kda
25 kda

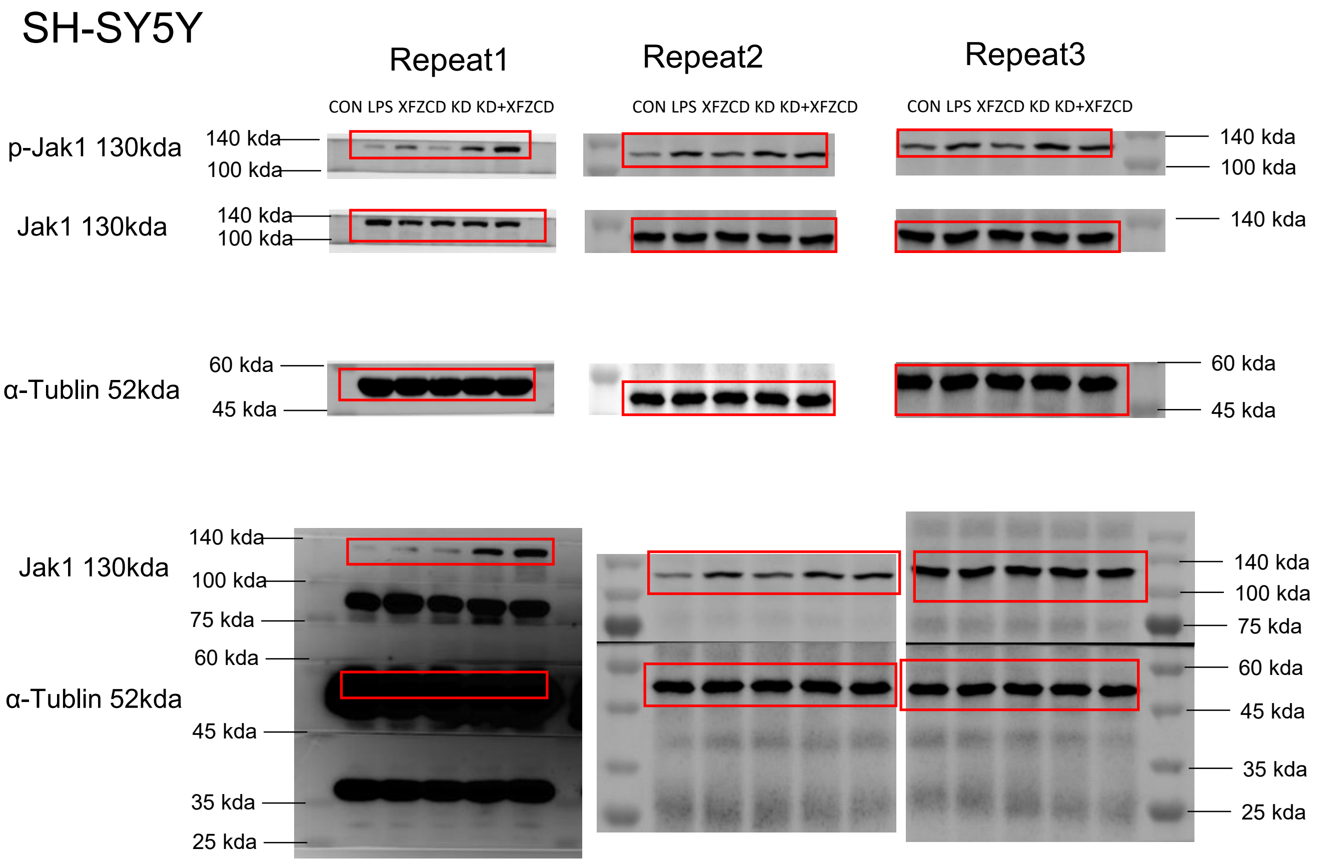
SH-SY5Y
Repeat1
Repeat2
Repeat3
CON LPS XFZCD KD KD+XFZCD
CON LPS XFZCD KD KD+XFZCD
CON LPS XFZCD KD KD+XFZCD
p-Jak1 130kda
Jak1 130kda
α-Tublin 52kda
Jak1 130kda
α-Tublin 52kda
140 kda
100 kda
75 kda
60 kda
45 kda
35 kda
25 kda
